# Skeletal Muscle Immunometabolism in Women With Polycystic Ovary Syndrome: A Meta-Analysis

**DOI:** 10.3389/fphys.2020.573505

**Published:** 2020-10-22

**Authors:** Maria Manti, Elisabet Stener-Victorin, Anna Benrick

**Affiliations:** ^1^Department of Physiology and Pharmacology, Karolinska Institutet, Stockholm, Sweden; ^2^Department of Physiology, Sahlgrenska Academy, University of Gothenburg, Gothenburg, Sweden; ^3^School of Health Sciences, University of Skövde, Skövde, Sweden

**Keywords:** PCOS, transcriptomics, gene expression, immunometabolism, meta-analysis, skeletal muscle

## Abstract

Polycystic ovary syndrome (PCOS) is an endocrine and metabolic disorder affecting up to 15% of women at reproductive age. The main features of PCOS are hyperandrogenism and irregular menstrual cycles together with metabolic dysfunctions including hyperinsulinemia and insulin resistance and a 4-fold increased risk of developing type 2 diabetes. Despite the high prevalence the pathophysiology of the syndrome is unclear. Insulin resistance in women with PCOS likely affect the skeletal muscle and recently it was demonstrated that changes in DNA methylation affects the gene expression in skeletal muscle that in part can explain their metabolic abnormalities. The objective of this work was to combine gene expression array data from different datasets to improve statistical power and thereby identify novel biomarkers that can be further explored. In this narrative review, we performed a meta-analysis of skeletal muscle arrays available from Gene Expression Omnibus and from publications. The eligibility criteria were published articles in English, and baseline (no treatment) skeletal muscle samples from women with PCOS and controls. The R package Metafor was used for integration of the datasets. One hundred and fourteen unique transcripts were differentially expressed in skeletal muscle from women with PCOS vs. controls (*q* < 0.05), 87% of these transcripts have not been previously identified as altered in PCOS muscle. *ING2, CDKAL1*, and *AKTIP* had the largest differential increase in expression, and *TSHZ2, FKBP2*, and *OCEL1* had the largest decrease in expression. Two genes, *IRX3* and *CDKAL1* were consistently upregulated (*q* < 0.05) in the individual analyses and meta-analysis. Based on the meta-analysis, we identified several dysregulated immunometabolic pathways as a part of the molecular mechanisms of insulin resistance in the skeletal muscle of women with PCOS. The transcriptomic data need to be verified by functional analyses as well as proteomics to advance our understanding of PCOS specific insulin resistance in skeletal muscle.

## Introduction

Polycystic ovary syndrome (PCOS) is a common endocrine disorder among women of reproductive age, which is characterized by biochemical or clinical hyperandrogenism, irregular cyclicity and polycystic ovarian morphology (Norman et al., 2007; Teede et al., 2018). Metabolic dysfunction is evident in women with PCOS, and manifests as impaired glucose homeostasis, dyslipidemia and abdominal obesity (Azziz et al., 2016). Women with PCOS have epigenetic and transcriptional changes in skeletal muscle that, in part, can explain the metabolic abnormalities seen in these women (Skov et al., 2007; Nilsson et al., 2018). In addition, defects in early insulin signaling in combination with fibrosis in the skeletal muscle, likely contribute to insulin resistance in women with PCOS (Stepto et al., 2020).

There is an increasing body of publications supporting a role for both innate and adaptive immunity in response to changes in metabolic status. This new field of immunometabolism builds on evidence for activation of immune-derived signals in metabolically relevant tissues and explores how immune cells support tissues to adapt to environmental challenges (Man et al., 2017). Adipose tissue is one of the most explored tissues in the field, and the expression and activation of various immune cell types and anti-inflammatory cytokines have been studied in both lean and obese states (Ferrante, 2013; Man et al., 2017). Skeletal muscle is another key metabolically active organ considering that it is the most effective organ for insulin-stimulated glucose uptake in the body (~80%) (Thiebaud et al., 1982). It also plays an important role in the development and progression of type 2 diabetes (Petersen and Shulman, 2018). However, there is scarce knowledge on how immune cells support the metabolic function in the skeletal muscle and on the role of inflammation in modulating skeletal muscle metabolism. The possible impact of immune cells on whole body glucose uptake and insulin sensitivity is a relatively recent appreciation.

Insulin resistance is a central feature of PCOS, with 30–40% of women with the syndrome having glucose intolerance, and 10% of them develop type 2 diabetes before the age of 40 (Legro et al., 1999; Rubin et al., 2017). Clinical studies exploring the molecular pathways of PCOS-insulin resistance in the skeletal muscle are limited. We have previously performed a wide-scale transcriptomic analysis in the skeletal muscle of women with PCOS and reported that many enriched pathways were involved in immune function or immune diseases (Nilsson et al., 2018). Due to the limited sample size of our study and the need to better understand the skeletal muscle metabolism in PCOS, we aim here to perform an integrated meta-analysis of the three available gene expression arrays in skeletal muscle of overweight/obese women with impaired glucose homeostasis. We further explore whether immunometabolism pathways are dysregulated in the skeletal muscle of these women.

## Methods

### Selection of Studies

We collected array studies including skeletal muscle from women with and without PCOS published between January 1999 and August 21, 2020, by performing a computerized search using PubMed, Omnibus GEO and Array Express. No review protocol exists but the following key words were used: human, skeletal muscle, polycystic ovary syndrome, PCOS, array, and gene expression. The eligibility criteria were; published articles, published in English, and baseline samples (no treatment). Data was collected from the GEO database (Skov et al., 2007, 2008) and from our own publication (Nilsson et al., 2018). The analysis is reported according to the PRISMA checklist (Moher et al., 2009).

### Meta-Analysis

The datasets were collected from two different microarray platforms, namely Affymetrix and Illumina. Affymetrix data was downloaded from NCBI GEO (GSE8157 and GSE6798), and normalized with methods described in Skov et al. (2007). The CEL-files and additional files were downloaded using the GEOquery R package. In order to check which samples were present in both GSE8157 and GSE6798, since some samples was used in both studies (Skov et al., 2007, 2008) but with different names, we used the R package DupChecker to compare the CEL-files. The Illumina data was background corrected and normalized using NormExp method in the R package limma (Shi et al., 2010). A fixed-effect meta-analysis model, with inverse-variance method, was used to estimate the possible associations between gene expression and PCOS. The Q statistic from the meta-analysis was used to identify the presence of heterogeneity. To account for multiple testing and control for false positives, the *p*-values from the meta-analysis were adjusted, using the Benjamini-Hochberg procedure. The robust genes, showing statistically significant association with PCOS, were identified as genes with false discovery rate (FDR) *q* < 0.05, and are presented as effect size. A heat map for all significant genes (*q* < 0.05) and a forest plot for the two most robust genes were produced. All analyses and plots were performed using the R statistical programming software [Version 4.0.2]. The meta-analysis was conducted using the rma() function in the Metafor package (Viechtbauer, 2010), the heatmap was produced using the heatmap.2() function in the gplot package and the FDR was estimated using the stats package.

### Pathway Analyses

We applied gene set enrichment analysis (GSEA. https://www.gsea-msigdb.org/gsea/index.jsp) to the expression array data using Broad Institute Gene sets database. All transcripts were used and ranked according to the t statistics in a *t-*test. The GSEA considered pathways with 5–5,000 transcripts. Finally, we performed a Gene Ontology (GO) enrichment analysis using the DAVID web resource (Huang da et al., 2009) to identify those biological functions and processes that are shared by the differentially expressed genes (*q* < 0.05). Gene groups were subjected to Functional Annotation Clustering using the GOTERM_BP_FAT and KEGG_PATHWAY categories, and annotation clusters with enrichment score at least 1.3, corresponding to *P* < 0.05 (Huang da et al., 2009), were considered enriched.

## Results

### Meta-Analysis of mRNA Expression in Skeletal Muscle From Women With PCOS and Controls

A total of 24 studies were identified, 12 were assessed for eligibility, and three studies were included in the meta-analysis as they fulfilled the eligibility criteria; published articles, published in English, and baseline samples (no treatment) (Skov et al., 2007, 2008; Nilsson et al., 2018). A PRISMA flow diagram is included in Supplementary Figure 1 (Prisma checklist in Supplementary Table 1). Only 3 samples in GSE8157 were exclusive for this study, while the others were also included in GSE6798. This generated a total number of 27 controls and 36 PCOS cases in the meta-analysis. Table 1 shows the most relevant clinical characteristics of the patients from each cohort. All women were over-weight or obese (mean BMI > 30 kg/m^2^), and premenopausal. Women with PCOS were hyperandrogenic with metabolic aberration and insulin resistance. PCOS was diagnosed according to 2 criteria; irregular periods with cycle length > 35 days, and biochemical or clinical signs of hyperandrogenism in Skov et al. (2007, 2008), and according to Rotterdam criteria in Nilsson et al. (2018) fulfilling 2 of 3 criteria; ultrasound-verified polycystic ovaries, oligomenorrhea/amenorrhea, and/or clinical signs of hyperandrogenism.

**Table 1:** Clinical characteristics of the patients in the 3 cohorts included in the meta-analysis.

**Characteristic**	**Skov et al. (2007) Control/PCOS**	**Skov et al. (2008) Control/PCOS**	**Nilsson et al. (2018) Control/PCOS**
Total subjects	13/16	13/10	14/17
Included subjects	13/16	0/3	14/17
Age (years)	34.7 ± 2.0/30.8 ± 1.8	34.7 ± 2.0/30.3 ± 2.1	29.7 ± 5.85/31.2 ± 5.4
BMI (kg/m^2^)	34.0 ± 1.8/34.1 ± 1.1	34.0 ± 1.8/33.2 ± 0.9	30.2 ± 3.6/31.3 ± 4.3
Insulin (pmol/L)	43 ± 4/116 ± 16*	51 ± 6/125 ± 23*	63 ± 27/89 ± 55
Glucose (mmol/L)	5.5 ± 0.1/5.5 ± 0.1	5.6 ± 0.1/5.9 ± 0.2	5.1 ± 0.4/4.9 ± 0.3
Free testosterone (nmol/L)	0.021 ± 0.000/0.059 ± 0.010*	0.025 ± 0.003/0.053 ± 0.009*	0.015 ± 0.005/0.029 ± 0.019*
LH/FSH ratio	0.68 ± 0.06/1.55 ± 0.17*	No data	1.18 ± 0.84/1.80 ± 1.13

In total, 27 controls and 36 women with PCOS were included. ^*^P < 0.05 PCOS vs. control subjects in each cohort. Data presented as mean ± SEM in Skov et al. (2007, 2008), and as mean ± SD in Nilsson et al. (2018).

**Figure 1:**
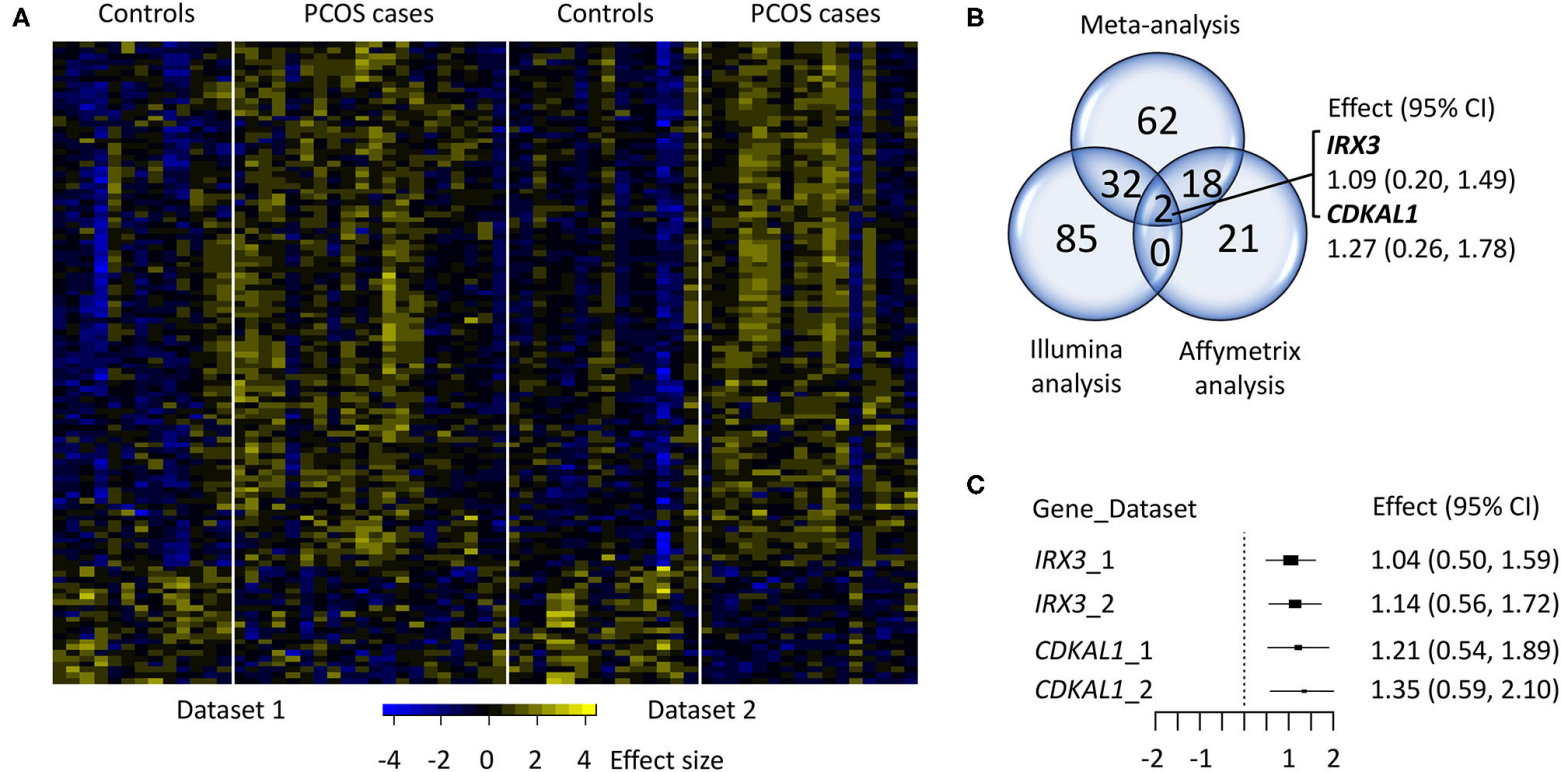
Comparison of individual analysis in dataset 1 (Skov et al., 2007, 2008) and 2 (Nilsson et al., 2018). **(A)** Heat map of the 114 differentially expressed genes (*q* < 0.05), **(B)** Venn diagram of the individual analysis; Illumina, Affymetrix, and integrated meta-analysis (*q* < 0.05). **(C)** Forest plot of the effect size of the two genes, *IRX3*, and *CDKAL1*, consistently upregulated in the three studies.

In total, of the 8,351 analyzed transcripts, 1,228 were differentially expressed in skeletal muscle from women with PCOS vs. controls (*P* < 0.05). After correction for multiple testing by FDR (5%, *q* < 0.05), 114 unique transcripts remained significant (Figure 1A, Table 2). In total, 82% of the transcripts were upregulated and 18% were downregulated in women with PCOS. The three genes with the largest differential increase in expression were *ING2, CDKAL1*, and *AKTIP*, and the three genes with the largest differential decrease in expression were *TSHZ2, FKBP2*, and *OCEL1*. Ninety-nine of the 114 identified transcripts have not been previously shown to be altered in skeletal muscle arrays from women with PCOS (Skov et al., 2007, 2008; Nilsson et al., 2018) (marked in bold in Table 2).

**Table 2:** Differentially expressed transcripts in skeletal muscle from women with PCOS vs. controls in this meta-analysis (*q* < 0.05).

**Gene**	**ES**	***q***	**Gene**	**ES**	***q***	**Gene**	**ES**	***q***
*ING2*	1.32	0.002	***MYLK4***	0.93	0.004	***AES***	0.78	0.050
***CDKAL1***	1.27	0.002	***USP53***	0.93	0.017	***INPP5A***	0.77	0.047
*AKTIP*	1.17	0.020	***NFIA***	0.92	0.033	***MOCS2***	0.75	0.030
*RAPH1*	1.16	0.004	***STXBP3***	0.92	0.019	***UBR3***	0.75	0.030
***UBC***	1.15	0.040	***RRM2B***	0.92	0.013	***ANXA7***	0.75	0.044
*ADK*	1.13	0.004	***PSMC4***	0.92	0.050	***NEDD1***	0.74	0.031
*HFE2*	1.13	0.004	***PPP1R3B***	0.92	0.009	***COL4A3***	0.74	0.038
*CSPP1*	1.11	0.007	***RBM24***	0.91	0.017	***FAM129A***	0.74	0.033
*SCP2*	1.11	0.005	***PLCL1***	0.91	0.017	***MSRB3***	0.74	0.049
*MAPKAP1*	1.11	0.004	***HIST1H2AC***	0.90	0.011	***C1ORF43***	0.74	0.047
***CACNB1***	1.10	0.013	***VCL***	0.89	0.042	***ENSA***	0.74	0.030
***ABLIM1***	1.10	0.005	***KRR1***	0.89	0.042	***CLK1***	0.73	0.047
***IRX3***	1.09	0.001	***NGDN***	0.89	0.042	***BPGM***	0.73	0.050
***UNC13B***	1.09	0.012	***FBXW7***	0.88	0.010	***FBXO32***	0.72	0.047
***SHISA2***	1.08	0.002	***MCL1***	0.88	0.022	***CALML6***	0.70	0.017
***HSF2***	1.08	0.012	***PGK1***	0.87	0.023	***MLF1***	0.69	0.048
*DUSP13*	1.07	0.026	***PHKG1***	0.87	0.009	***LRRC3B***	0.68	0.030
***IREB2***	1.07	0.005	*PDE4DIP*	0.87	0.017	***LPL***	−0.71	0.046
***SYNC***	1.05	0.005	***EGF***	0.86	0.030	***HMOX1***	−0.76	0.038
***ADPRHL1***	1.04	0.008	***GRB14***	0.86	0.007	***ADM***	−0.77	0.049
***ATP2A1***	1.02	0.002	***CTNNB1***	0.85	0.030	***UCP2***	−0.78	0.026
***MORC3***	1.01	0.004	***SERPINE2***	0.85	0.019	***MT2A***	−0.81	0.030
***RCN2***	1.01	0.017	***FAM184B***	0.85	0.038	***SKAP2***	−0.81	0.030
***ATP2C1***	1.00	0.005	***TSPAN3***	0.85	0.018	***HSPB6***	−0.82	0.030
***HOMER1***	1.00	0.007	***CHAF1B***	0.85	0.031	***MT1E***	−0.85	0.031
***OAT***	1.00	0.008	***PTBP2***	0.84	0.033	***PC***	−0.86	0.033
***MSTN***	0.99	0.002	*PITX2*	0.84	0.045	***ATOH8***	−0.89	0.030
***CTBP1***	0.98	0.010	***ARMC8***	0.84	0.049	*NBPF20*	−0.89	0.045
***ATP5G2***	0.97	0.030	***NCKAP1***	0.83	0.012	***SNX21***	−0.89	0.033
***EGFLAM***	0.96	0.019	***PDZRN3***	0.83	0.035	***AGPAT9***	−0.89	0.030
***TSC22D3***	0.96	0.030	***ACVR1***	0.83	0.021	***LDHB***	−0.92	0.008
***TMEM182***	0.96	0.017	***TRDN***	0.83	0.013	***IFITM3***	−0.93	0.046
***PRR16***	0.95	0.004	***USP46***	0.81	0.047	*THY1*	−0.95	0.017
***H2AFY***	0.95	0.030	***GPD2***	0.81	0.043	***RSPO3***	−0.96	0.012
***FRMD6***	0.95	0.009	***RAN***	0.81	0.040	***BST2***	−1.08	0.016
***CLTCL1***	0.93	0.030	***ALDOA***	0.79	0.030	***OCEL1***	−1.15	0.013
***DCAF6***	0.93	0.042	***RB1***	0.79	0.041	*FKBP2*	−1.26	0.013
*ZBTB16*	0.93	0.020	***PPTC7***	0.79	0.042	***TSHZ2***	−1.29	0.006

*Transcripts not previously shown to be altered in PCOS muscle arrays (Skov et al., 2007, 2008; Nilsson et al., 2018) are marked in bold. ES, Enrichment score*.

In Nilsson et al. (2018), we have previously investigated the overlap between the differentially expressed genes identified in that study (*p* < 0.05) and those found in Skov et al. (2007) (*q* < 0.1), and found four transcripts (i.e., *RAPH1, INO80D, PPIE*, and *AKTIP*) that were upregulated in both studies. Two of these genes, *RAPH1* and *AKTIP* were significant at *q* < 0.05 (effect size 1.16 and 1.17, respectively) and *PPIE* at *q* < 0.1 (effect size 0.86) in the integrated meta-analysis, while *INO80D* was not significantly altered. The meta-analysis findings were also compared with those obtained by individual analyses in both datasets (Supplementary Table 2) to evaluate bias and reproducibility across the microarray studies. As a result, 34 genes identified in the Illumina data set were also highlighted by our meta-analysis, whereas 20 genes were shared with the Affymetrix data set (Figure 1B). Two of these genes, *IRX3* (effect size 1.09) and *CDKAL1* (effect size 1.27) were consistently upregulated (*q* < 0.05), by the three studies (Affymetrix, Illumina and integrated meta-analysis) (Figure 1C).

### Gene Set Enrichment Analysis and Pathway Analysis

**Figure 2:**
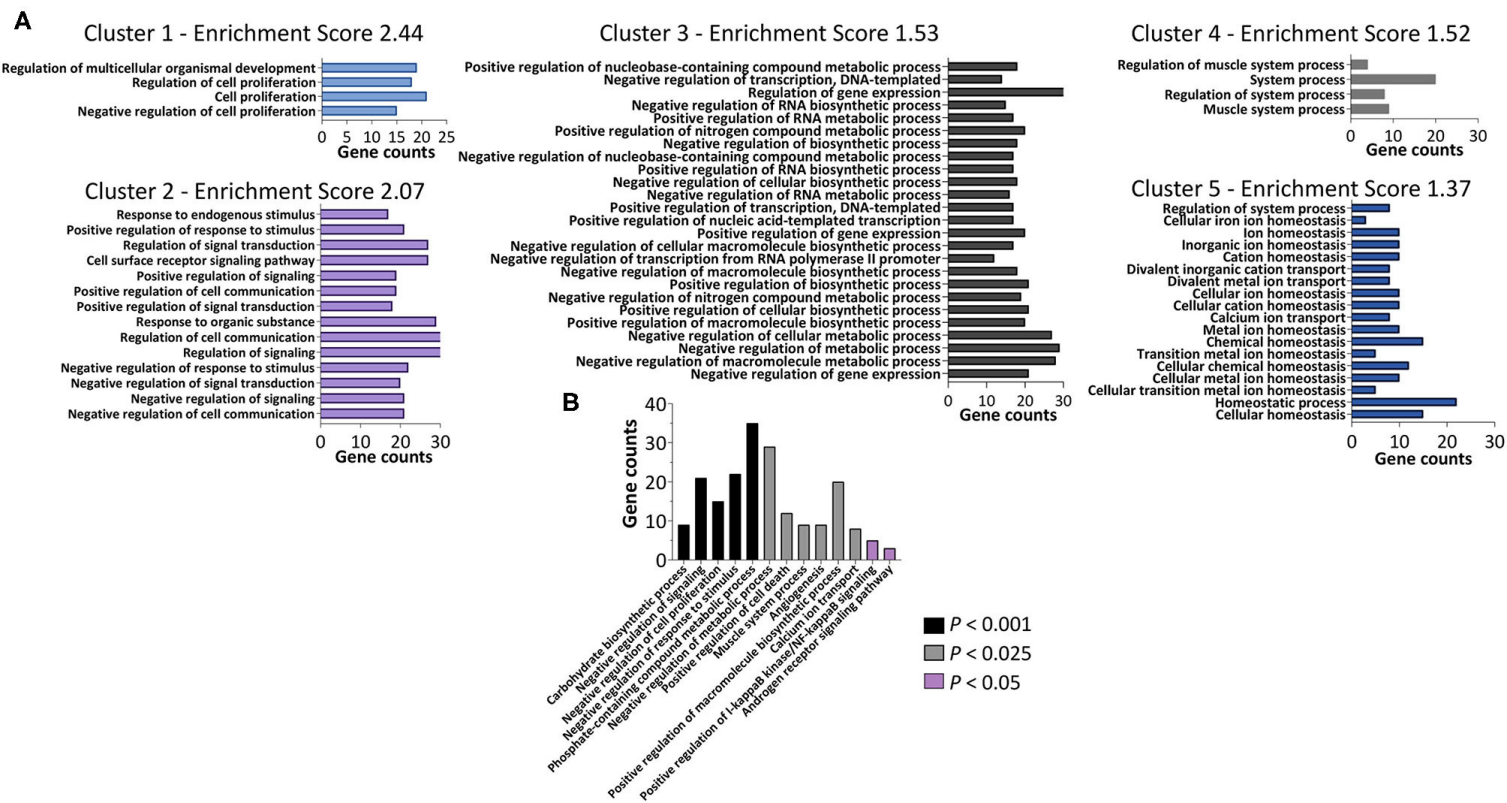
Functional annotation clustering determined using DAVID Bioinformatics Resources with respect to the 114 differently expressed genes (*q* < 0.05) in the skeletal muscle from women with PCOS compared with controls. **(A)** The representative groups with an enrichment score of 1.3 or above are presented. The x-axis represents the number of genes, while the y-axis represents the ontology categories. **(B)** Selected GO terms of biological processes enriched by the differently expressed genes (*P* < 0.05).

Next, we tested whether sets of biologically-related genes were altered in women with PCOS compared with controls. According to the GSEA, there were significant (*q* < 0.05) gene expression alterations in 9 downregulated pathways in women with PCOS (Table 3). The majority of these downregulated pathways are involved in inflammatory/immune response while myogenesis and MYC signaling, a transcription factor for growth-related genes for glycolysis and mitochondrial metabolism in T cells (Wang et al., 2011), were upregulated (*p* < 0.05). Finally, GO analysis including the 114 differently expressed transcripts (*q* < 0.05) showed 205 enriched signaling pathways (Figure 2A, Supplementary Table 3). Genes involved in metabolic and carbohydrate biosynthetic processes, positive regulation of I-kappaB kinase/NF-kappaB signaling, and negative regulation of cell proliferation and positive regulation of cell death appeared in the enriched pathways (Figure 2B). Muscle system processes and calcium ion transport were also enriched together with the androgen receptor signaling pathway (Figure 2B, Supplementary Table 3). All three genes in the androgen receptor signaling pathway; *RAN, RB1*, and *CTNNB1*, were significantly upregulated in women with PCOS compared to controls (*q* < 0.05, Table 2).

**Table 3:** Significantly differentially expressed gene sets in skeletal muscle from women with PCOS compared with controls (GSEA, *q* ≦0.25).

**Gene set name**	**Size**	**ES**	**NES**	***p***	***q***
Upregulated enriched gene sets					
MYC TARGETS V1	165	0.37	1.71	0.000	0.023
SPERMATOGENESIS	46	0.45	1.71	0.000	0.043
G2M CHECKPOINT	99	0.34	1.47	0.019	0.109
HEME METABOLISM	124	0.32	1.43	0.013	0.124
MYOGENESIS	163	0.29	1.34	0.035	0.20
Downregulated enriched gene sets					
INTERFERON ALPHA RESPONSE	67	−0.57	−2.45	0.000	0.000
INTERFERON GAMMA RESPONSE	127	−0.44	−2.14	0.000	0.001
CHOLESTEROL HOMEOSTASIS	50	−0.49	−2.00	0.000	0.001
XENOBIOTIC METABOLISM	117	−0.42	−2.00	0.000	0.001
COAGULATION	66	−0.45	−1.94	0.000	0.001
EPITHELIAL MESENCHYMAL TRANSITION	130	−0.39	−1.89	0.000	0.002
APOPTOSIS	114	−0.32	−1.54	0.003	0.048
IL2 STAT5 SIGNALING	117	−0.32	−1.52	0.000	0.047
APICAL JUNCTION	111	−0.31	−1.51	0.003	0.047
COMPLEMENT	106	−0.31	−1.45	0.008	0.067
HEDGEHOG SIGNALING	16	−0.43	−1.31	0.125	0.19
INFLAMMATORY RESPONSE	86	−0.29	−1.30	0.070	0.20
HYPOXIA	134	−0.26	−1.27	0.063	0.21
TNFA SIGNALING VIA NFKB	116	−0.26	−1.24	0.083	0.25
P53 PATHWAY	136	−0.25	−1.24	0.065	0.23
ADIPOGENESIS	164	−0.24	−1.23	0.078	0.22
ALLOGRAFT REJECTION	87	−0.27	−1.22	0.130	0.24
IL6 JAK STAT3 SIGNALING	40	−0.31	−1.20	0.192	0.25
ANGIOGENESIS	22	−0.35	−1.20	0.208	0.24

*The enrichment score (ES) was calculated for each gene set, the primary outcome of GSEA. NES, Enrichment score normalized for differences in gene set size*.

## Discussion

Taking an integrated meta-analysis approach, this study showed an overall downregulation of transcripts involved in the immune system and several alterations in metabolic pathways in skeletal muscle of women with PCOS. Previous data suggests that insulin signaling defects in skeletal muscle partly account for the insulin resistance in obese women with PCOS. However, our data indicate that additional molecular mechanisms are involved in the PCOS-insulin resistance in skeletal muscle. Fibrosis might be one key player (Stepto et al., 2020), together with immunometabolic alterations as supported by our data.

Fibrosis in skeletal muscle can impair muscle function and affect muscle fiber regeneration after injury (Delaney et al., 2017; Mahdy, 2019). Tissue fibrosis is often initiated and maintained through TGF-beta signaling and has been suggested to be associated with insulin resistance and steatosis in PCOS (Petta et al., 2017; Stepto et al., 2019). This association is supported by a recent paper, investigating TGF-beta ligand induced fibrosis in obese women with PCOS (Stepto et al., 2020). Moreover, we have previously shown an enrichment of extracellular matrix signaling pathways and a decreased collagen gene expression in our previous study (Nilsson et al., 2018), supporting a link between dysregulated TGF-beta signaling and extracellular matrix deposition with insulin resistance in PCOS. Surprisingly, fibrosis, extracellular matrix-, or TGF-beta signaling pathways were not enriched in women with PCOS in this meta-analysis. However, the most upregulated gene in PCOS muscle, inhibitor of growth family member 2 (*ING2*), promotes TGF-beta-induced transcription and cellular responses (Sarker et al., 2008), which leave the issue open for further investigation.

We have previously shown that the majority of the significant gene expression pathways (GSEA) in women with PCOS were involved in immune responses or were related to immune diseases (Nilsson et al., 2018). A distinct pattern was a downregulation of human leukocyte antigen (HLA) genes in women with PCOS. This finding was not validated in this meta-analysis, although two HLA genes were downregulated (*p* < 0.05), but they did not survive FDR <5%. However, here we show a dysregulated expression of many genes involved in immune pathways, including a downregulation of gene sets associated with inflammatory response, interferon alpha and gamma response and the proinflammatory cytokines interleukin-2, interleukin-6 and TNF-alpha. Among the dysregulated genes, *ADK* and *BST2* are thought to play a role in the immune system, identified as having anti-inflammatory and anti-viral actions, respectively (Douglas et al., 2010; Boison, 2013). *BST2* was one of the top downregulated genes, and is known to be an interferon-inducible gene, and to play a role in innate immunity (Douglas et al., 2010). *FKBP2* was also one of the top downregulated genes, which plays a role in immunoregulation by binding the immunosuppressive compound rapamycin that inhibits mTOR signaling (Hendrickson et al., 1993). This is in line with the downregulation of enriched gene sets associated with interferon alpha and gamma responses. Looking into the most upregulated genes, *ADK* is a phosphotransferase that converts the purine ribonucleotide adenosine into 5′-adenosine-monophosphate (Boison, 2013). This reaction controls largely the adenosine tone, and consequently affects a wide range of functions. One of the functions that ADK and adenosine exert, is the regulation of the immune system function. Adenosine is evidenced to be generated as a result of stress response in damaged tissues and to display immunosuppressive properties (Hasko and Cronstein, 2004). A downregulation of inflammatory pathways in skeletal muscle is in contrast to the chronic low-grade pro-inflammatory state in adipose tissue (Dimitriadis et al., 2016). There is a lack of conclusive evidence, but androgens likely have direct effects on the adipose tissue resident immune cells (Huang et al., 2013). Changes in adipocyte function impair the secretion of adipokines, leading to decreased adiponectin secretion (Manneras-Holm et al., 2011), which promotes susceptibility to low grade inflammation. Despite the different immune responses in skeletal muscle and adipose tissue, it is evident that immune function is impaired in women with PCOS.

Inflammatory and metabolic pathways in skeletal muscle are closely tied to cell signaling and differentiation, which leads to tissue specific adaptations in response to different stimuli e.g., androgens, obesity and exercise. The GSEA showed an upregulated myogenesis and the GO showed an enrichment of genes involved in negative regulation of cell proliferation, positive regulation of cell death, and negative regulation of response to stimuli, supporting skeletal muscle specific adaptations in response to PCOS. Furthermore, genes involved in MYC signaling were the most upregulated enriched gene set in the GSEA. MYC is a transcription factor that drives metabolic reprogramming in activated, primary T lymphocytes, and this may be one of the key targets responsible for metabolic reprogramming in response to PCOS conditions (Wang et al., 2011). As expected, many metabolic pathways were found to be enriched in the GO, with a negative regulation of the majority of the metabolic processes. *ALDOA*, encoding aldolase, was upregulated in women with PCOS and seems to be a key gene involved in many carbohydrate metabolic processes. Aldolase is an insulin stimulated glycolytic enzyme that catalyzes the conversion of fructose-1,6-bisphosphate during glycolysis, but it is also involved in actin remodeling, possibly modulating both metabolic and structural tissue adaptations (Hu et al., 2016).

Lastly, two genes were consistently upregulated in all three studies, *IRX3* and *CDKAL1*. Both genes have been associated with body mass index and type 2 diabetes in genome-wide association studies (Steinthorsdottir et al., 2007; Ragvin et al., 2010; Yengo et al., 2018). *IRX3* is a transcription and neuronal progenitor factor, and acts as a regulator of energy metabolism. It is shown to be controlled by a non-coding region of the fat mass and obesity-associated (FTO) gene, which is one of the strongest obesity-associated genes found in humans (Frayling et al., 2007). Many studies have investigated the role of BMI-associated variants in women with PCOS (Jones and Goodarzi, 2016). The results are controversial, but a meta-analysis showed that *FTO* has an increased effect on the BMI of women with PCOS, but it is not a PCOS susceptibility locus (Wojciechowski et al., 2012). Moreover, data for *CDKAL1* and *FTO* suggest that polymorphisms of these genes are not associated with insulin resistance or insulin secretory capacity in Asian women with PCOS (Kim et al., 2012).

The interpretation of this meta-analysis is limited by a fairly low number of women with PCOS and controls (*n* < 40/group), the inclusion of a single ethnicity (Caucasian), and a mix of PCOS phenotypes (Rotterdam criteria). Further, our findings cannot distinguish whether the identified dysregulated pathways are responses to a stimulus or the causal effectors. Another limitation is the fact that we cannot identify the gene expression changes within specific target cells i.e., myocytes, as the muscle biopsy is a bulk of many different cell types and structures, e.g., immune cells, vessels and connective tissue.

## Conclusion

Clinical studies and transcriptomics data clearly demonstrate that molecular dysfunction in skeletal muscle contributes to insulin resistance in women with PCOS. Intracellular insulin-signaling pathways, mitochondrial function and fat oxidation, with a possible contribution of reduced adiponectin levels, have all been in focus for the mechanism of insulin resistance in PCOS (Stepto et al., 2019). Here, we identify 99 genes not previously shown to be altered in PCOS muscle, two of them consistently upregulated in all three studies. We show that immunometabolism can be added to the list of dysfunctional pathways and present genes with large effect size that warrant further investigation. However, gene expression does not always translate to alterations in biological function and data on total and phosphorylated protein levels in skeletal muscle is limited. Therefore, functional studies and proteomics analysis is needed to validate and advance our understanding of insulin resistance in skeletal muscle in these women.

## Data Availability Statement

All datasets presented in this study are included in the article/Supplementary Material.

## Author Contributions

MM, ES-V, and AB designed the study and contributed to the interpretation of data. MM and AB performed data analyses and statistical analyses, and wrote the manuscript. All authors critically revised and approved the manuscript. All authors contributed to the article and approved the submitted version.

## Conflict of Interest

The authors declare that the research was conducted in the absence of any commercial or financial relationships that could be construed as a potential conflict of interest.

## Supplementary Material

Supplementary Figure 1 PRISMA flow chart.

Supplementary Table 1 PRISMA check list.

Supplementary Table 2 Individual analyses of transcripts in dataset 1 (Skov et al., 2007, 2008) and 2 (Nilsson et al., 2018) (*P* < 0.01).

Supplementary Table 3 Overrepresentation analysis using gene ontology (GO) on the 114 genes differentially expressed in skeletal muscle from women with PCOS compared with controls (*q* < 0.05).
