# Supplementary material for: Skeletal Muscle Immunometabolism in Women With Polycystic Ovary Syndrome: A Meta-Analysis
Source: Front Physiol. 2020 Oct 22;11:573505. doi: 10.3389/fphys.2020.573505 (PMC7642984; doi:10.3389/fphys.2020.573505)
Supplement: Supplementary Figure 1 — PRISMA flow chart. [file Data_Sheet_3.PDF]

Supplemental Figure 1

PRISMA 2009 Flow Diagram

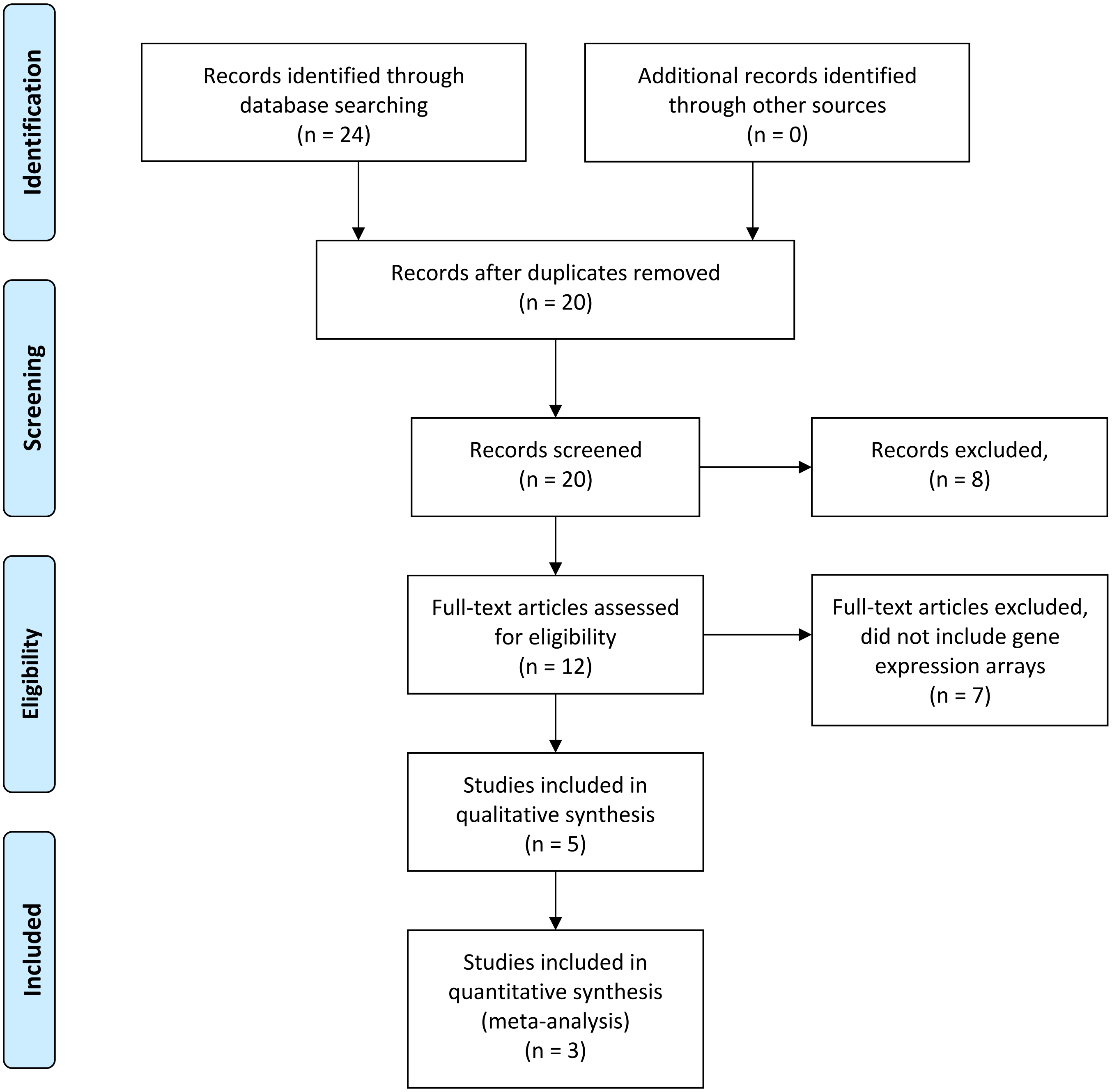

From: Moher D, Liberati A, Tetzlaff J, Altman DG, The PRISMA Group (2009). *P*referred *R*eporting *I*tems for *S*ystematic Reviews and *M*eta-*A*nalyses: The PRISMA Statement. PLoS Med 6(7): e1000097. doi:10.1371/journal.pmed1000097

For more information, visit www.prisma-statement.org.
