# Supplementary material for: Skeletal Muscle Immunometabolism in Women With Polycystic Ovary Syndrome: A Meta-Analysis
Source: Front Physiol. 2020 Oct 22;11:573505. doi: 10.3389/fphys.2020.573505 (PMC7642984; doi:10.3389/fphys.2020.573505)
Supplement: Supplementary Table 2 — Individual analyses of transcripts in dataset 1 (Skov et al., 2007, 2008) and 2 (Nilsson et al., 2018) (P < 0.01). [file Data_Sheet_2.PDF]

Supplemental Table 2. Individual analyses of transcripts in Dataset 1 (Skov *et al*., 2007; Skov *et al*., 2008) and 2 (Nilsson *et al*., 2018) ($p < 0.01$)

	Dataset 1		Dataset 2	
**Gene**	**Effect Size**	***p***	**Effect Size**	***p***
SEPT2	0,50	0,148	1,15	**0,004**
SEPT11	1,17	**0,002**	-0,61	0,028
A2M	0,51	0,132	-1,01	**0,006**
ABCA3	1,11	**0,007**	-0,14	0,728
ABCC9	-0,16	0,572	1,30	**0,002**
ABHD3	-0,31	0,299	1,17	**0,004**
ABLIM1	1,38	**0,001**	0,93	**0,003**
ABLIM3	0,17	0,650	-0,89	**0,008**
ACOT11	-1,00	**0,005**	-0,23	0,362
ACP1	0,60	0,140	0,79	**0,008**
ACP2	0,52	0,215	-1,15	**0,005**
ACTN3	0,77	**0,001**	0,13	0,553
ACTN4	0,41	0,378	-0,88	**0,008**
ACVR1	1,08	**0,001**	0,60	0,044
ADAR	0,26	0,496	-1,12	**0,006**
ADCY1	-0,24	0,410	1,19	**0,001**
ADK	0,93	**0,005**	1,40	**0,000**
ADM	-0,56	0,074	-0,99	**0,002**
ADPRHL1	0,88	**0,009**	1,24	**0,001**
ADSL	0,32	0,375	1,19	**0,008**
ADSSL1	0,33	0,414	1,00	**0,001**
AEBP1	0,22	0,474	-1,23	**0,000**
AES	0,74	0,041	0,82	**0,006**
AFAP1L1	0,79	**0,006**	0,31	0,337
AGPAT2	0,09	0,726	-0,85	**0,002**
AGPAT9	-0,82	**0,009**	-1,01	**0,009**
AHCYL1	1,06	**0,007**	0,03	0,943
AHNAK	1,40	**0,000**	0,04	0,891
AIFM1	0,31	0,417	0,99	**0,003**
AK1	0,99	**0,004**	0,51	0,118
AKIRIN1	0,17	0,534	0,82	**0,009**
AKNA	-0,54	0,213	-1,04	**0,004**
AKR1B10	0,37	0,229	1,04	**0,001**
AKTIP	1,54	**0,001**	0,88	0,032
ALDH6A1	-0,98	**0,001**	0,07	0,797
ALDH7A1	-1,19	**0,004**	-0,22	0,506
ALDOA	0,98	**0,007**	0,69	**0,009**
ALDOC	0,29	0,548	-0,90	**0,002**
ALKBH5	0,94	**0,009**	0,61	0,116
ALPK2	-1,31	**0,000**	-0,05	0,880
AMACR	-0,37	0,295	1,31	**0,001**
AMD1	0,14	0,634	1,52	**0,000**
AMFR	1,18	**0,000**	0,10	0,711
ANKRD1	-0,14	0,504	-0,73	**0,006**

ANKRD26	-0,65	0,039	1,35	**0,001**
ANKRD33	0,14	0,773	-1,80	**0,000**
ANXA2P1	-0,24	0,510	-1,03	**0,000**
ANXA7	0,34	0,280	1,12	**0,000**
AOC3	0,12	0,613	-0,71	**0,007**
AP2M1	0,93	0,028	-1,17	**0,003**
APH1A	0,52	0,135	-1,12	**0,009**
APLNR	0,13	0,695	-0,93	**0,007**
APOE	0,66	0,018	-0,81	**0,003**
AQP4	-0,54	0,030	0,88	**0,002**
ARF5	0,23	0,588	-1,67	**0,001**
ARHGDIA	0,70	0,041	-1,21	**0,001**
ARHGEF11	1,17	**0,009**	-0,49	0,262
ARHGEF2	-0,29	0,444	-0,95	**0,006**
ARID2	-0,01	0,961	1,06	**0,008**
ARID5B	-0,17	0,538	0,90	**0,008**
ARL6IP4	0,17	0,649	-1,10	**0,006**
ARMC8	0,42	0,211	1,33	**0,000**
ARPC2	1,23	**0,002**	-0,17	0,708
ARRDC3	-0,06	0,800	0,95	**0,007**
ASB11	0,00	0,995	1,07	**0,001**
ASB2	0,90	**0,002**	-0,49	0,142
ATF5	0,19	0,520	-0,84	**0,009**
ATG4B	-0,03	0,947	1,16	**0,000**
ATL2	0,31	0,364	1,00	**0,003**
ATN1	0,29	0,382	-1,31	**0,001**
ATOH8	-0,79	0,032	-0,96	**0,003**
ATOX1	0,66	0,139	-1,39	**0,000**
ATP13A1	0,60	0,168	-1,12	**0,003**
ATP1A1	0,27	0,545	-1,05	**0,007**
ATP2A1	1,04	**0,001**	1,01	**0,001**
ATP2C1	0,80	**0,008**	1,27	**0,000**
ATP5C1	-1,02	0,014	0,97	**0,008**
ATP5F1	-0,35	0,334	0,99	**0,002**
ATP5G2	0,99	0,013	0,95	**0,008**
ATP6V0D1	1,45	**0,003**	-0,91	0,041
ATP8B4	0,07	0,875	-0,81	**0,006**
ATPAF1	1,01	**0,004**	0,09	0,821
BBS2	-0,03	0,917	1,26	**0,001**
BCCIP	-0,54	0,169	1,04	**0,004**
BCLAF1	-0,13	0,609	1,04	**0,003**
BIN1	1,15	**0,001**	0,23	0,536
BLOC1S1	-0,05	0,894	-1,05	**0,006**
BSDC1	0,50	0,158	-1,78	**0,000**
BST2	-0,82	0,039	-1,33	**0,000**
BTBD1	0,01	0,986	1,18	**0,000**
BTBD3	0,55	0,061	1,16	**0,003**
BTF3	0,06	0,875	1,07	**0,005**

C11ORF58	-0,01	0,973	0,95	**0,004**
C11ORF74	-1,08	**0,009**	0,02	0,965
C12ORF5	-0,36	0,181	0,97	**0,003**
C16ORF58	0,09	0,837	-1,20	**0,003**
C19ORF10	-0,39	0,392	-1,01	**0,007**
C1ORF43	0,75	0,033	0,74	**0,007**
C1QTNF1	-0,21	0,516	-0,88	**0,003**
C1S	0,61	0,084	-0,91	**0,005**
C2CD2	-0,22	0,485	-0,74	**0,006**
C3ORF38	-0,19	0,507	1,10	**0,002**
C9ORF142	-0,11	0,690	-1,51	**0,002**
CA3	0,14	0,702	0,81	**0,005**
CACNB1	1,49	**0,000**	0,82	0,022
CALB2	0,26	0,518	-0,90	**0,000**
CALML6	0,84	**0,001**	0,58	0,022
CAMK2D	0,38	0,167	0,93	**0,007**
CAMK2G	0,33	0,409	1,17	**0,002**
CAND2	1,37	**0,003**	-0,14	0,747
CAPN5	-0,09	0,820	-1,32	**0,003**
CAPN7	0,17	0,584	1,12	**0,003**
CAPRIN1	0,23	0,464	1,05	**0,006**
CARHSP1	0,60	0,315	-1,05	**0,004**
CASP4	-0,02	0,960	-1,17	**0,005**
CASQ1	1,55	**0,003**	0,24	0,479
CBR3	-0,22	0,563	-0,99	**0,009**
CCBE1	-1,02	**0,008**	0,18	0,536
CCDC69	0,89	**0,004**	-0,16	0,623
CCNC	0,35	0,304	1,04	**0,001**
CCND1	0,50	0,143	-0,74	**0,004**
CCT6A	0,43	0,190	1,01	**0,001**
CCT7	-0,03	0,947	1,34	**0,000**
CD248	-0,11	0,800	-0,96	**0,001**
CD276	0,70	0,104	-1,85	**0,000**
CD36	-0,51	0,052	0,77	**0,002**
CD74	-0,21	0,548	-0,94	**0,004**
CD81	0,80	0,047	-0,96	**0,003**
CD99	0,73	0,063	-1,36	**0,001**
CDC42EP5	0,43	0,384	-1,30	**0,002**
CDIPT	-1,15	**0,008**	-0,32	0,473
CDKAL1	1,21	**0,000**	1,35	**0,000**
CDKN1C	-1,20	**0,001**	-0,20	0,553
CEBPA	0,33	0,413	-0,80	**0,002**
CEBPD	-0,79	**0,005**	0,50	0,121
CECR1	-0,17	0,621	-0,91	**0,005**
CEP350	-0,54	0,057	0,90	**0,008**
CFH	-0,01	0,967	-0,97	**0,005**
CFL2	0,15	0,684	1,47	**0,000**
CHAF1B	1,18	**0,002**	0,65	0,029

CHCHD7	-0,12	0,724	1,32	**0,002**
CHMP5	0,51	0,134	0,83	**0,006**
CHST15	0,75	**0,008**	-0,04	0,879
CHSY1	0,09	0,773	-1,28	**0,005**
CHUK	1,15	**0,003**	-0,02	0,971
CIB2	1,10	**0,007**	0,06	0,851
CIDEA	-0,18	0,649	-0,78	**0,001**
CIDEC	0,27	0,354	-0,90	**0,000**
CIDECP	0,27	0,616	-0,66	**0,009**
CKB	-0,88	**0,003**	-0,26	0,306
CLASP2	-0,48	0,103	1,08	**0,000**
CLDN5	0,26	0,395	-1,06	**0,002**
CLEC2D	0,18	0,684	0,98	**0,002**
CLIC1	0,00	0,989	-1,09	**0,001**
CLIP4	-0,11	0,714	0,91	**0,002**
CLK1	0,44	0,119	1,15	**0,001**
CLTCL1	0,58	0,112	1,28	**0,000**
CMAS	1,08	**0,005**	0,36	0,317
CMBL	0,39	0,224	0,99	**0,005**
CMIP	-0,32	0,445	-0,89	**0,007**
COBLL1	0,06	0,843	-0,81	**0,003**
COL18A1	0,57	0,070	-1,18	**0,003**
COL1A1	0,63	0,012	-1,33	**0,000**
COL3A1	0,54	0,039	-1,02	**0,001**
COL4A3	0,66	0,022	0,84	**0,006**
COL6A1	0,67	0,034	-1,30	**0,000**
COL6A2	0,78	0,013	-1,60	**0,000**
CORO1C	0,15	0,589	-0,88	**0,007**
COX6A1	-0,19	0,631	-0,79	**0,007**
CRABP2	-0,01	0,982	-1,05	**0,008**
CRADD	0,41	0,357	1,06	**0,005**
CRIM1	-0,43	0,119	0,97	**0,001**
CRIP1	-0,12	0,673	-0,91	**0,003**
CSDE1	0,19	0,651	1,68	**0,000**
CSNK1A1	0,41	0,203	1,31	**0,000**
CSPP1	0,83	**0,007**	1,79	**0,000**
CST3	-0,46	0,136	-1,44	**0,001**
CSTB	-0,28	0,547	-0,97	**0,006**
CTBP1	0,98	**0,004**	0,97	**0,003**
CTNNB1	0,72	0,035	0,97	**0,002**
CTSK	-0,43	0,229	-0,87	**0,007**
CTSZ	-0,35	0,328	-1,28	**0,000**
CYB5A	0,40	0,186	-0,74	**0,008**
CYB5R1	-0,48	0,277	-1,02	**0,008**
CYB5R4	0,42	0,269	1,36	**0,001**
CYBA	0,05	0,881	-0,83	**0,009**
CYGB	-0,35	0,289	-1,42	**0,001**
CYR61	0,26	0,390	-0,86	**0,002**

CYYR1	-0,88	**0,009**	-0,31	0,390
DAPK2	-0,19	0,641	-0,95	**0,008**
DCAF16	0,41	0,148	1,56	**0,001**
DCAF6	0,65	0,162	1,08	**0,001**
DCUN1D1	0,53	0,047	0,82	**0,008**
DDIT4L	1,07	**0,001**	-0,01	0,980
DDX17	0,08	0,736	1,11	**0,001**
DDX19B	-0,02	0,956	1,38	**0,000**
DDX21	0,07	0,832	1,29	**0,003**
DDX46	-0,12	0,750	1,38	**0,001**
DFNA5	-1,02	**0,003**	-0,37	0,177
DGAT2	-0,21	0,529	-0,88	**0,002**
DHCR24	1,01	**0,006**	0,11	0,722
DHDDS	0,27	0,423	-0,83	**0,003**
DHRS7B	0,98	**0,007**	0,17	0,657
DICER1	0,34	0,230	1,14	**0,001**
DNAJB6	0,12	0,746	1,15	**0,000**
DNAJC12	-1,25	**0,000**	-0,04	0,902
DNAJC24	-0,32	0,411	0,97	**0,010**
DNASE1L1	0,04	0,922	1,53	**0,000**
DOK5	0,57	0,084	0,83	**0,005**
DPT	0,39	0,124	-0,83	**0,003**
DR1	0,11	0,708	1,06	**0,003**
DUSP13	0,59	0,190	1,40	**0,000**
DUSP14	0,44	0,321	-1,06	**0,001**
DUSP3	0,93	**0,003**	0,09	0,810
DVL1	0,09	0,801	0,99	**0,006**
DYRK1A	-0,07	0,843	1,44	**0,000**
ECM2	-0,03	0,902	-0,79	**0,004**
EDC4	1,40	**0,005**	-0,01	0,982
EEF1A1	0,25	0,475	-0,88	**0,006**
EGF	0,68	0,021	1,18	**0,002**
EGFL6	0,22	0,392	-0,69	**0,004**
EGFLAM	0,82	0,061	1,03	**0,001**
EIF3F	0,77	**0,009**	0,74	0,046
EIF4E2	1,42	**0,000**	-0,16	0,708
EIF4E3	0,27	0,334	0,94	**0,009**
EIF4G2	-0,44	0,224	1,16	**0,000**
EIF4G3	1,07	**0,002**	-0,31	0,376
EIF5A	0,84	**0,003**	0,24	0,523
ELMO2	1,14	**0,005**	0,17	0,694
ENPEP	-0,80	**0,007**	-0,20	0,578
ENPP2	-0,08	0,734	-0,76	**0,006**
ENSA	0,80	**0,007**	0,69	0,013
EPHX1	0,02	0,939	-0,99	**0,001**
EPS15L1	-0,03	0,916	1,36	**0,002**
ERGIC1	1,01	**0,006**	0,13	0,629
ERP29	0,72	0,152	-1,40	**0,001**

ESRRG	-0,77	**0,007**	-0,34	0,217
ETS1	0,11	0,701	-0,93	**0,006**
EXOC1	0,01	0,966	1,24	**0,003**
EXOC4	0,53	0,144	1,59	**0,000**
EXT1	-0,55	0,208	-1,31	**0,002**
EYA1	0,52	0,114	0,91	**0,007**
EYA4	0,22	0,492	1,29	**0,001**
FABP4	-0,15	0,537	-0,76	**0,003**
FAF1	0,23	0,419	1,28	**0,000**
FAH	-0,73	0,069	-0,83	**0,008**
FAM107B	-0,19	0,541	-0,82	**0,006**
FAM129A	0,67	0,018	0,82	**0,006**
FAM134A	0,48	0,188	1,16	**0,008**
FAM184B	0,65	0,041	1,11	**0,002**
FASN	0,16	0,591	-0,87	**0,000**
FASTK	-0,05	0,906	0,92	**0,008**
FASTKD2	-0,19	0,541	0,99	**0,008**
FAT1	-0,34	0,263	-0,91	**0,002**
FBLN1	-0,11	0,740	-0,92	**0,005**
FBXL5	0,67	0,035	0,85	**0,009**
FBXO3	0,09	0,764	1,11	**0,001**
FBXO32	0,50	0,099	0,90	**0,002**
FBXO34	0,33	0,383	1,09	**0,003**
FBXW7	0,57	0,055	1,21	**0,000**
FDX1	-0,20	0,527	1,33	**0,003**
FGD5	0,27	0,435	-1,40	**0,001**
FILIP1	0,10	0,736	1,11	**0,005**
FKBP2	-0,73	0,138	-1,62	**0,000**
FLI1	0,16	0,602	-1,28	**0,005**
FLJ36848	0,00	0,990	1,22	**0,000**
FLNB	-0,33	0,383	-0,96	**0,010**
FMOD	-0,26	0,525	-0,73	**0,007**
FNTA	0,32	0,404	0,92	**0,002**
FOXO3	0,05	0,858	0,87	**0,007**
FRMD6	1,33	**0,000**	0,55	0,089
FRZB	1,07	**0,000**	0,06	0,793
FSTL3	0,10	0,823	-0,74	**0,008**
FXR1	-0,23	0,406	1,19	**0,000**
FXYD5	0,23	0,569	-0,96	**0,005**
FYTTD1	0,52	0,084	0,99	**0,003**
G0S2	1,15	**0,000**	-0,25	0,372
G3BP2	0,32	0,210	0,98	**0,001**
GAB2	1,58	**0,003**	-0,41	0,189
GATM	-0,58	0,032	0,84	**0,003**
GCDH	-1,32	**0,002**	-0,62	0,078
GCLC	-1,01	**0,007**	-0,53	0,175
GFM2	-0,31	0,360	1,21	**0,001**
GMFB	-0,20	0,442	-1,01	**0,007**

GNL3	0,00	0,995	1,07	**0,005**
GOLGA4	0,03	0,942	0,98	**0,005**
GPD1	0,93	**0,003**	-0,61	0,048
GPD1L	-0,55	0,105	1,09	**0,004**
GPD2	0,93	**0,003**	0,67	0,054
GPN3	0,31	0,366	1,03	**0,002**
GPX1	0,00	0,996	-1,21	**0,001**
GPX3	-0,51	0,089	-0,89	**0,007**
GRB14	0,90	**0,001**	0,81	**0,005**
GRM2	-0,22	0,537	1,57	**0,001**
GSR	1,04	**0,008**	0,09	0,809
GSS	0,05	0,888	-1,37	**0,003**
GSTO1	-1,39	**0,002**	-0,36	0,287
GTF2H2B	-0,14	0,622	1,07	**0,002**
GTF2I	0,40	0,268	1,21	**0,000**
GYG2	0,10	0,705	-0,83	**0,001**
H2AFJ	1,05	**0,004**	-0,19	0,531
H2AFY	0,99	0,011	0,92	**0,009**
HAT1	-0,04	0,909	0,98	**0,001**
HBB	0,78	**0,003**	-0,48	0,087
HCCS	-0,22	0,472	1,12	**0,002**
HECTD1	-0,31	0,228	0,84	**0,010**
HFE2	0,77	0,041	1,41	**0,000**
HINT3	0,41	0,108	0,90	**0,007**
HIST1H2AC	0,90	**0,006**	0,90	**0,002**
HLA-DMB	-0,60	0,210	-0,91	**0,005**
HLA-F	0,44	0,199	-1,13	**0,000**
HLA-G	0,60	0,093	-1,01	**0,003**
HMGB2	-1,08	**0,001**	-0,38	0,299
HMOX1	-0,64	0,033	-0,89	**0,004**
HNRNPK	0,41	0,287	1,04	**0,001**
HOMER1	1,09	**0,001**	0,90	**0,007**
HOXB6	-0,27	0,474	0,94	**0,007**
HOXB7	0,35	0,342	-0,81	**0,010**
HS6ST2	1,00	**0,000**	0,29	0,298
HSDL2	0,97	**0,007**	0,17	0,629
HSF2	0,99	**0,003**	1,24	**0,005**
HSPA12A	0,04	0,903	-0,95	**0,001**
HSPB6	-1,12	**0,001**	-0,59	0,049
HTRA1	0,26	0,431	-0,91	**0,002**
ID2	-0,38	0,155	-1,07	**0,002**
IDE	-0,26	0,452	1,09	**0,002**
IDI2	-0,94	**0,001**	-0,06	0,839
IER2	1,03	**0,009**	-0,02	0,954
IER3	-0,88	**0,005**	-0,17	0,497
IFI27L2	-0,35	0,304	-1,28	**0,002**
IFITM2	-0,19	0,583	-1,16	**0,002**
IFITM3	-0,60	0,137	-1,20	**0,001**

IGFBP3	-0,13	0,732	-0,86	**0,004**
IGFBP4	-0,01	0,979	-1,25	**0,000**
IGFBP5	0,87	**0,004**	0,00	0,997
IL17D	-0,93	**0,001**	-0,05	0,889
ILF3	1,08	**0,004**	-0,35	0,377
ING2	1,08	**0,001**	1,71	**0,000**
ING4	-0,09	0,853	-1,05	**0,009**
INPP5A	0,94	**0,001**	0,50	0,171
IPO7	-0,56	0,093	1,01	**0,003**
IREB2	1,45	**0,000**	0,77	0,017
IRF2BP2	-0,85	**0,005**	0,39	0,243
IRF8	-0,49	0,264	-0,78	**0,010**
IRS2	-1,11	**0,003**	0,73	0,026
IRX3	1,04	**0,000**	1,14	**0,000**
ISCA2	-1,21	**0,004**	0,01	0,978
ISG15	-0,29	0,297	-0,92	**0,004**
ISY1	0,59	0,153	0,90	**0,003**
JAK1	-0,25	0,413	0,97	**0,001**
JDP2	1,12	**0,004**	0,17	0,660
KCNS3	1,07	**0,005**	0,70	0,048
KIAA1715	0,81	**0,007**	0,37	0,308
KIF1B	0,80	**0,006**	0,25	0,378
KIF5B	0,14	0,652	0,92	**0,003**
KLF10	0,09	0,722	1,39	**0,000**
KLF15	-1,03	**0,005**	0,69	0,021
KLF2	0,14	0,629	-0,86	**0,006**
KLF9	0,03	0,930	1,19	**0,001**
KLHL20	0,32	0,300	1,54	**0,000**
KLHL23	0,13	0,739	1,27	**0,005**
KRR1	0,60	0,064	1,38	**0,001**
KRT31	1,25	**0,003**	0,13	0,672
KRTCAP2	0,17	0,687	-1,24	**0,009**
LAMA4	0,20	0,449	-0,91	**0,002**
LAMB2	1,06	**0,009**	-0,64	0,089
LDHB	-1,38	**0,000**	-0,57	0,048
LDHD	-1,01	**0,003**	-0,56	0,062
LDLR	0,10	0,739	-0,77	**0,006**
LEP	0,28	0,219	-0,91	**0,000**
LFNG	-0,25	0,506	-1,16	**0,000**
LGALS3	-0,58	0,121	-0,89	**0,006**
LGALS3BP	0,40	0,353	-0,94	**0,003**
LIMCH1	1,28	**0,001**	0,03	0,932
LIPA	-0,17	0,622	-0,82	**0,009**
LIPE	-0,24	0,497	-0,69	**0,007**
LMF2	0,13	0,804	-0,96	**0,007**
LMNA	0,88	**0,007**	-0,96	0,029
LMO2	0,96	**0,003**	0,29	0,358
LMOD1	0,79	**0,008**	0,42	0,154

LMOD2	-1,44	**0,002**	-0,04	0,882
LMOD3	-0,82	**0,009**	-0,05	0,875
LOC10019098	0,22	0,391	1,46	**0,000**
LOC151121	0,90	**0,001**	0,35	0,234
LOC285556	0,79	**0,009**	0,21	0,458
LOC399491	-0,02	0,959	1,13	**0,009**
LOC401052	-0,24	0,546	-0,85	**0,005**
LONRF2	1,00	**0,001**	0,17	0,689
LPL	-1,56	**0,000**	-0,28	0,279
LPP	-0,77	**0,005**	-0,38	0,276
LRP5	0,09	0,791	-1,12	**0,001**
LRRC3B	0,72	**0,004**	0,63	0,020
LRRC41	1,41	**0,001**	0,04	0,917
LRRFIP2	1,23	**0,002**	0,14	0,654
LSM12	0,22	0,494	0,91	**0,005**
LTBP4	0,25	0,401	-1,21	**0,000**
LTBR	0,24	0,367	-1,43	**0,000**
LYPLA1	0,43	0,180	1,10	**0,001**
MAD2L1BP	-0,74	0,108	0,90	**0,005**
MAFF	-0,96	**0,000**	-0,08	0,781
MAP2K6	-0,78	0,020	1,41	**0,000**
MAP4K3	0,44	0,121	1,09	**0,001**
MAP7D1	1,25	**0,001**	-0,01	0,986
MAPKAP1	0,76	0,011	1,73	**0,000**
MAPKAPK2	0,74	**0,010**	0,44	0,226
MAPKAPK3	1,28	**0,004**	0,24	0,535
MAPRE3	0,23	0,392	-0,95	**0,007**
MAT2B	-0,18	0,572	1,53	**0,000**
MATR3	0,01	0,968	1,21	**0,000**
MBP	0,18	0,544	0,99	**0,005**
MCEE	-1,15	**0,006**	0,83	0,019
MCL1	0,75	0,019	1,02	**0,002**
MCOLN1	0,50	0,304	-1,38	**0,001**
MDFIC	-0,20	0,382	0,77	**0,007**
MDK	-0,18	0,553	-0,98	**0,009**
ME1	1,30	**0,001**	0,06	0,862
MED27	-0,41	0,255	1,24	**0,000**
MEOX1	-0,49	0,112	-1,12	**0,003**
MEOX2	0,10	0,693	-0,90	**0,009**
MFAP4	0,36	0,354	-1,05	**0,001**
MFSD11	1,22	**0,003**	0,18	0,533
MGLL	0,31	0,388	-0,89	**0,006**
MGP	-0,21	0,418	-0,75	**0,005**
MGST1	0,05	0,818	-0,87	**0,000**
MLF1	1,23	**0,000**	0,29	0,275
MLKL	-0,37	0,267	-0,95	**0,007**
MMACHC	-1,49	**0,005**	-0,04	0,916
MOCS2	0,74	0,021	0,76	**0,004**

MORC3	0,85	**0,007**	1,19	**0,000**
MORC4	1,18	**0,001**	-0,23	0,595
MORF4L2	-0,58	0,057	0,94	**0,002**
MRAP	1,06	0,018	-0,70	**0,005**
MRAS	1,14	**0,003**	-0,42	0,194
MRPL45	-0,22	0,625	0,89	**0,003**
MRPS22	-1,28	**0,009**	0,36	0,450
MRS2	0,39	0,246	0,95	**0,007**
MSN	0,27	0,376	-1,23	**0,001**
MSRB3	0,53	0,079	0,96	**0,002**
MSTN	1,28	**0,000**	0,74	**0,008**
MT1E	-0,98	**0,004**	-0,74	0,022
MT2A	-0,82	**0,010**	-0,80	0,010
MTHFD2	0,93	**0,002**	0,18	0,546
MTUS1	0,25	0,459	1,34	**0,002**
MX1	-0,17	0,620	-1,12	**0,002**
MXRA5	0,23	0,368	-0,84	**0,003**
MYBPC1	0,51	0,545	0,96	**0,001**
MYBPC2	1,23	**0,000**	0,30	0,279
MYF6	0,51	0,136	-0,66	**0,007**
MYH1	1,06	**0,000**	0,19	0,415
MYH9	-0,70	0,037	-0,88	**0,007**
MYL1	0,52	0,485	0,87	**0,005**
MYL5	0,83	**0,007**	0,56	0,038
MYL9	-0,04	0,908	-0,80	**0,007**
MYLK3	-0,81	**0,004**	0,88	0,012
MYLK4	1,27	**0,000**	0,67	0,012
MYO18B	0,80	**0,008**	0,30	0,343
MYOF	-0,17	0,556	-0,89	**0,007**
MYOZ2	-1,03	**0,001**	0,53	0,056
MYRIP	0,46	0,113	0,96	**0,005**
NADSYN1	-0,10	0,694	-1,32	**0,002**
NAMPT	0,19	0,474	1,34	**0,000**
NANOS1	1,47	**0,000**	-0,24	0,462
NBEA	-0,31	0,304	1,11	**0,005**
NBL1	-0,14	0,716	-1,02	**0,005**
NBPF20	-0,33	0,383	-1,37	**0,000**
NBPF8	-0,36	0,323	-1,19	**0,004**
NCKAP1	0,59	0,035	1,12	**0,000**
NDRG2	0,83	**0,002**	-0,33	0,377
NDRG3	1,19	**0,003**	0,50	0,181
NDUFA3	-1,27	**0,008**	-0,40	0,391
NEDD1	0,39	0,149	1,24	**0,000**
NEDD4	0,05	0,866	1,27	**0,001**
NEXN	0,04	0,920	1,14	**0,006**
NFIA	1,13	**0,001**	0,58	0,169
NFIL3	-0,90	**0,010**	0,28	0,411
NGDN	0,68	0,063	1,08	**0,002**

NGRN	0,13	0,679	0,91	**0,002**
NIN	0,87	**0,007**	0,55	0,089
NIPSNAP3A	0,91	**0,003**	0,36	0,393
NME4	-0,24	0,523	-1,44	**0,000**
NMNAT1	0,07	0,816	1,07	**0,005**
NMT1	-0,14	0,733	-1,15	**0,004**
NNAT	-0,07	0,870	-0,72	**0,007**
NOSTRIN	-0,68	0,072	-0,98	**0,007**
NPEPPS	-0,13	0,662	1,04	**0,004**
NPY6R	-0,88	**0,005**	1,07	**0,001**
NQO1	0,26	0,436	-0,92	**0,001**
NR1H3	-0,36	0,362	-0,85	**0,004**
NRIP1	-0,22	0,331	1,09	**0,005**
NUDT6	-0,15	0,626	0,91	**0,009**
NUP35	0,14	0,655	0,90	**0,007**
NUP62	0,63	0,068	1,24	**0,003**
NUPR1	-0,05	0,867	-0,83	**0,009**
OAS2	-0,04	0,890	-0,93	**0,006**
OAT	1,14	**0,002**	0,90	**0,004**
OCEL1	-1,08	0,013	-1,19	**0,001**
OIP5	0,84	**0,008**	0,31	0,322
OLFM1	0,06	0,798	-0,79	**0,006**
OPTN	-0,18	0,651	1,06	**0,009**
OSBPL7	1,32	**0,000**	0,01	0,985
OSBPL9	-0,25	0,429	1,22	**0,001**
OSTF1	1,35	**0,003**	-0,11	0,729
OXCT1	-0,74	**0,007**	-0,17	0,574
P4HB	0,85	**0,003**	-0,41	0,286
PAIP2	-0,42	0,365	1,09	**0,005**
PALM	0,57	0,096	-0,82	**0,005**
PAM	-0,96	**0,008**	-0,08	0,844
PAQR9	-0,81	**0,005**	-0,41	0,181
PARN	1,23	**0,010**	-0,08	0,846
PARVB	0,45	0,099	1,26	**0,001**
PC	-0,67	0,103	-0,96	**0,001**
PCDH18	-0,17	0,595	-0,78	**0,005**
PCMTD1	-0,97	**0,004**	0,04	0,918
PCOLCE	0,08	0,842	-0,97	**0,006**
PCOLCE2	0,31	0,176	-0,69	**0,006**
PDE4DIP	0,49	0,081	1,51	**0,000**
PDGFB	-0,34	0,274	-1,12	**0,005**
PDGFRB	-0,11	0,763	-1,58	**0,000**
PDHA1	-0,89	**0,008**	0,63	0,085
PDK4	-0,66	**0,006**	0,48	0,042
PDLIM3	0,88	**0,003**	0,04	0,909
PDLIM5	0,28	0,284	1,15	**0,000**
PDPK1	0,23	0,534	1,10	**0,002**
PDSS2	-1,43	**0,000**	0,04	0,907

PDXK	0,44	0,196	-0,69	**0,009**
PDZRN3	1,06	**0,002**	0,63	0,049
PEAR1	0,03	0,935	-1,31	**0,005**
PEMT	0,14	0,755	-0,96	**0,007**
PEX1	0,34	0,311	1,19	**0,006**
PEX7	-0,86	0,038	0,89	**0,008**
PGK1	0,90	**0,004**	0,84	0,016
PGLS	1,11	0,010	-1,05	**0,006**
PGM2L1	0,57	0,062	0,98	**0,004**
PHGDH	0,37	0,391	-0,91	**0,001**
PHKG1	0,82	**0,002**	0,94	**0,004**
PHLDA3	0,43	0,271	-0,82	**0,005**
PHLDB2	0,28	0,236	-0,92	**0,003**
PHTF2	0,29	0,209	0,79	**0,005**
PHYH	-0,18	0,622	0,84	**0,002**
PIK3CB	0,50	0,129	1,04	**0,003**
PIP5K1C	-0,03	0,952	-1,10	**0,003**
PITX2	0,49	0,147	1,20	**0,001**
PLAC9	0,39	0,290	-0,76	**0,007**
PLAU	-0,14	0,690	-1,37	**0,001**
PLCL1	0,89	**0,010**	0,92	**0,004**
PLEKHB2	0,32	0,333	1,55	**0,000**
PLEKHO2	0,97	0,044	-1,19	**0,001**
PLTP	-0,02	0,953	-0,87	**0,002**
PLXNB2	0,19	0,656	-0,91	**0,009**
PMEPA1	0,93	**0,005**	-0,17	0,549
PNPLA2	-0,52	0,127	-0,99	**0,002**
POLB	1,40	**0,001**	-0,16	0,621
POLR2L	-0,10	0,757	-1,51	**0,000**
PPAPDC3	1,27	**0,003**	-0,30	0,456
PPARG	0,04	0,926	-0,74	**0,004**
PPFIA1	0,57	0,110	1,08	**0,008**
PPL	0,05	0,874	-1,10	**0,004**
PPM1A	-0,24	0,374	1,41	**0,000**
PPM1B	-1,12	**0,003**	1,12	**0,002**
PPP1R1B	0,03	0,941	-0,89	**0,001**
PPP1R3A	-0,14	0,567	0,95	**0,002**
PPP1R3B	0,79	**0,007**	1,09	**0,001**
PPP2R2D	-0,29	0,472	1,55	**0,000**
PPP2R3A	0,49	0,142	0,97	**0,001**
PPP2R5B	-0,71	0,077	-0,99	**0,005**
PPP3R1	0,95	**0,001**	0,24	0,477
PPTC7	0,54	0,049	1,29	**0,001**
PRDM10	0,52	0,099	1,12	**0,008**
PRDX3	0,12	0,778	1,01	**0,001**
PREB	0,28	0,404	-0,92	**0,009**
PRKAG2	-0,89	**0,006**	-0,45	0,155
PRKAG3	0,90	**0,005**	0,11	0,701

PRKAR2A	0,30	0,426	1,13	**0,001**
PRKCDBP	-0,42	0,279	-0,98	**0,006**
PRNP	0,29	0,314	0,82	**0,010**
PROX1	-1,01	**0,003**	-0,11	0,741
PRPH2	1,01	**0,003**	0,02	0,962
PRPS1	0,91	**0,009**	0,46	0,315
PRR16	0,88	**0,001**	1,05	**0,001**
PSAP	1,00	**0,002**	-0,47	0,246
PSMA1	-0,30	0,478	0,98	**0,003**
PSMB7	-1,16	**0,006**	-0,05	0,909
PSMB8	0,53	0,162	-0,93	**0,005**
PSMC4	0,51	0,372	1,04	**0,001**
PSMD10	0,35	0,319	1,12	**0,002**
PTBP2	0,84	**0,007**	0,85	0,019
PTMS	-0,19	0,497	-1,06	**0,003**
PTPLB	0,17	0,500	-0,71	**0,007**
PTPRF	0,24	0,479	-0,69	**0,004**
PUF60	1,64	**0,003**	-0,37	0,245
PVALB	0,81	**0,002**	0,22	0,323
PVRL2	0,37	0,383	-0,96	**0,007**
PXMP2	-1,27	**0,005**	0,14	0,711
QKI	0,27	0,368	1,17	**0,000**
RAB3GAP1	1,36	**0,001**	0,49	0,302
RAB3IL1	-0,36	0,458	-1,26	**0,001**
RAB5C	0,10	0,771	-1,02	**0,007**
RABAC1	1,15	**0,008**	-0,20	0,569
RALGDS	0,48	0,226	-1,13	**0,004**
RAMP2	-0,05	0,901	-0,92	**0,006**
RAN	0,61	0,041	1,08	**0,002**
RAPH1	1,22	**0,000**	1,06	0,010
RARRES2	-0,12	0,703	-0,79	**0,002**
RARRES3	-0,21	0,529	-0,81	**0,008**
RASIP1	0,32	0,491	-1,07	**0,003**
RASSF1	0,38	0,394	-0,97	**0,004**
RB1	0,73	0,017	0,87	**0,010**
RBBP5	0,43	0,191	1,15	**0,006**
RBM18	0,02	0,952	0,94	**0,003**
RBM24	0,75	0,040	1,02	**0,001**
RBM25	-1,09	**0,001**	-0,47	0,264
RBM3	1,19	**0,000**	-0,18	0,554
RBM4	0,44	0,156	0,95	**0,003**
RBM41	-0,99	**0,010**	0,63	0,109
RBP4	0,35	0,124	-0,85	**0,001**
RBP7	0,60	0,071	-0,90	**0,002**
RCHY1	0,40	0,188	1,04	**0,004**
RCN2	0,80	0,032	1,20	**0,001**
RECK	0,02	0,945	-1,01	**0,006**
RELL1	-0,98	**0,003**	-0,03	0,928

RERE	0,88	**0,003**	0,46	0,222
RFXANK	-0,07	0,868	-1,31	**0,001**
RGL1	0,31	0,388	-1,10	**0,001**
RGS9BP	-0,11	0,739	0,92	**0,006**
RHOA	1,08	**0,005**	0,14	0,712
RHOBTB1	0,86	**0,006**	0,48	0,081
RHOT1	0,48	0,116	1,17	**0,003**
RIOK1	-0,55	0,100	1,56	**0,001**
RIOK3	0,26	0,393	1,29	**0,001**
RNASE1	-0,42	0,261	-0,93	**0,007**
RNASET2	0,42	0,170	-1,05	**0,006**
RNF135	1,38	**0,001**	-0,18	0,638
RNF166	1,16	**0,002**	0,11	0,792
RNFT1	0,15	0,578	0,94	**0,003**
RPL7	-1,33	**0,005**	0,05	0,909
RPLP0	0,61	0,079	0,84	**0,007**
RPRML	0,34	0,269	0,98	**0,001**
RPS6KA3	0,23	0,499	1,32	**0,000**
RPS6KA5	0,11	0,725	1,04	**0,007**
RRM2B	0,72	0,030	1,11	**0,000**
RSPO3	-1,08	**0,001**	-0,86	**0,010**
RSRC1	-0,31	0,300	1,05	**0,008**
RTN4	0,21	0,414	0,97	**0,003**
RWDD3	0,38	0,210	0,94	**0,006**
RYR3	-0,76	**0,006**	0,01	0,971
S100A11	0,36	0,233	-1,11	**0,002**
S100A13	1,05	**0,006**	-0,31	0,330
S100A8	0,62	**0,007**	-0,01	0,970
SAE1	1,10	**0,007**	0,32	0,427
SCAMP1	-0,71	0,034	1,09	**0,006**
SCD	0,08	0,671	-1,10	**0,000**
SCMH1	0,42	0,360	1,46	**0,001**
SCN1B	-0,43	0,302	1,07	**0,001**
SCP2	0,70	0,055	1,46	**0,000**
SDF4	0,37	0,307	-1,55	**0,001**
SEC24B	0,38	0,219	1,41	**0,002**
SECISBP2L	-0,97	**0,004**	-0,05	0,905
SELM	0,19	0,556	-1,21	**0,001**
SENP5	0,68	0,141	1,59	**0,001**
SERPINE2	1,06	**0,001**	0,67	0,025
SERPING1	-0,21	0,548	-1,06	**0,003**
SET	-0,34	0,416	1,23	**0,007**
SETD3	-0,96	0,015	1,13	**0,000**
SF3A3	0,74	**0,009**	0,15	0,616
SF3B1	-0,76	0,031	0,90	**0,008**
SF3B3	-0,34	0,465	-1,58	**0,001**
SGK1	0,73	**0,008**	-0,25	0,366
SH2D1B	1,12	**0,000**	0,26	0,310

SH3BP5L	-0,69	0,118	-0,83	**0,009**
SH3KBP1	-0,83	**0,005**	-0,19	0,557
SHISA2	1,20	**0,000**	0,95	**0,002**
SHISA5	0,28	0,495	-0,91	**0,010**
SIX1	-1,07	**0,007**	-0,30	0,395
SKAP2	-1,21	**0,000**	-0,43	0,165
SLC12A2	0,36	0,205	0,92	**0,003**
SLC25A20	0,28	0,440	1,00	**0,001**
SLC25A3	0,16	0,637	0,92	**0,003**
SLC25A6	0,57	0,089	-1,01	**0,008**
SLC27A3	0,68	0,111	-1,02	**0,004**
SLC29A1	0,83	**0,005**	-0,08	0,824
SLC29A4	-0,18	0,651	-0,77	**0,003**
SLC2A12	-1,07	**0,008**	-0,22	0,485
SLC2A3	-0,07	0,864	-0,89	**0,008**
SLC38A1	-1,17	**0,000**	0,05	0,856
SLC43A3	0,48	0,311	-1,22	**0,006**
SLC44A1	0,94	**0,006**	-0,29	0,420
SLC7A2	0,48	0,212	1,37	**0,001**
SMC3	-0,78	**0,007**	-0,05	0,885
SMTN	1,20	**0,002**	-0,19	0,575
SMTNL2	1,04	**0,005**	0,27	0,524
SNAP23	-0,73	0,021	0,92	**0,002**
SNRNP27	-0,13	0,729	1,05	**0,002**
SNRNP70	-0,14	0,632	-1,11	**0,004**
SNTB1	0,61	0,081	1,26	**0,002**
SNX21	-0,74	0,020	-1,14	**0,005**
SNX27	0,39	0,368	1,38	**0,003**
SNX5	0,17	0,613	1,19	**0,001**
SON	0,12	0,720	1,07	**0,005**
SOX6	-0,07	0,817	1,16	**0,004**
SPAG9	0,21	0,478	1,35	**0,000**
SPARC	1,33	**0,000**	-0,45	0,106
SPON1	-0,19	0,560	-0,83	**0,003**
SPRED1	0,04	0,917	-1,01	**0,005**
SPTAN1	-0,39	0,261	-1,21	**0,001**
SPTBN1	1,36	**0,001**	-0,04	0,902
SRP54	-0,16	0,622	1,27	**0,000**
SRP9	-0,32	0,519	1,57	**0,000**
SRPX	-0,10	0,711	-0,84	**0,002**
ST13	0,98	**0,006**	0,38	0,280
ST3GAL3	1,05	**0,001**	-0,53	0,147
ST6GALNAC6	-0,38	0,348	-0,98	**0,008**
STAB1	0,33	0,313	-1,32	**0,000**
STAU1	1,03	**0,007**	0,49	0,160
STK19	0,79	0,118	-1,30	**0,009**
STMN3	-0,52	0,218	-1,12	**0,004**
STOM	0,15	0,688	0,87	**0,009**

STRN3	0,37	0,203	1,33	**0,001**
STT3B	0,25	0,399	0,80	**0,006**
STXBP3	0,97	**0,002**	0,86	0,018
SUCLA2	0,10	0,800	0,92	**0,004**
SULF2	0,10	0,736	-1,30	**0,001**
SULT1A1	0,20	0,510	-1,22	**0,002**
SULT1A2	0,23	0,618	-1,32	**0,001**
SVIL	1,03	**0,006**	0,44	0,240
SYNC	1,29	**0,000**	0,88	**0,004**
SYNPO2	0,18	0,451	1,38	**0,000**
SYPL1	0,21	0,567	1,17	**0,000**
TAF13	1,04	**0,005**	0,04	0,905
TAGLN2	0,17	0,579	-1,14	**0,003**
TANK	-0,22	0,423	1,00	**0,009**
TBC1D8	0,00	0,998	1,10	**0,002**
TBC1D9	-0,26	0,331	-0,85	**0,006**
TBX1	0,39	0,114	0,85	**0,009**
TBX15	0,17	0,651	1,25	**0,001**
TCEA1	0,05	0,854	0,90	**0,002**
TCIRG1	0,49	0,203	-0,95	**0,006**
TEAD2	0,00	0,988	-1,01	**0,007**
TEAD4	1,21	**0,002**	0,11	0,778
TEF	0,27	0,499	-1,28	**0,005**
TF	0,71	0,053	-0,61	**0,010**
TFB2M	-0,51	0,157	1,24	**0,003**
TGFB1I1	0,01	0,960	-1,28	**0,002**
THAP1	-0,43	0,221	1,04	**0,001**
THRSP	0,45	0,055	-0,90	**0,000**
THY1	-0,33	0,325	-1,59	**0,000**
TIA1	0,30	0,274	0,99	**0,003**
TIAL1	0,30	0,336	1,34	**0,002**
TIMP1	0,34	0,292	-0,87	**0,008**
TIMP2	0,41	0,128	-1,03	**0,001**
TKT	0,40	0,169	-1,04	**0,000**
TM4SF1	-0,36	0,235	-1,19	**0,003**
TM6SF1	0,03	0,919	0,84	**0,003**
TMED9	0,08	0,837	-1,22	**0,009**
TMEM126A	-1,13	**0,008**	0,38	0,273
TMEM135	-0,25	0,291	-1,04	**0,004**
TMEM173	-0,01	0,980	-1,11	**0,006**
TMEM182	1,33	**0,000**	0,52	0,146
TMEM19	0,71	0,052	1,06	**0,005**
TMEM41A	0,06	0,872	1,40	**0,002**
TMEM54	-0,01	0,984	-1,15	**0,005**
TMOD1	1,25	**0,002**	0,62	0,119
TMTC1	-0,80	**0,006**	0,84	0,043
TNFRSF14	-0,03	0,936	-1,20	**0,003**
TNFRSF1B	0,27	0,410	-0,92	**0,009**

TNFRSF25	0,38	0,251	-0,77	**0,009**
TNNI2	1,28	**0,000**	0,00	0,991
TNNT1	-1,34	**0,006**	-0,53	0,153
TNNT3	1,34	**0,000**	0,31	0,310
TNPO1	0,66	0,032	-1,05	**0,009**
TNRC6B	0,28	0,307	1,57	**0,000**
TPD52L1	-1,50	**0,001**	0,03	0,937
TRAFD1	-0,37	0,224	-0,92	**0,009**
TRAK1	0,90	**0,004**	-0,44	0,243
TRDN	0,69	0,020	0,95	**0,001**
TRIM23	-0,01	0,973	1,34	**0,000**
TRIM24	-0,13	0,731	-0,73	**0,009**
TSC22D1	-0,29	0,340	0,93	**0,001**
TSC22D3	0,85	0,020	1,07	**0,004**
TSHZ2	-1,26	0,010	-1,31	**0,000**
TSNAX	-0,17	0,602	1,09	**0,001**
TSPAN13	-0,62	0,053	-1,02	**0,007**
TSPAN3	1,39	**0,001**	0,65	0,011
TSPAN4	0,49	0,145	-1,03	**0,003**
TTC7B	0,99	**0,003**	0,59	0,160
TUBA4A	0,95	**0,006**	-0,16	0,609
TUBGCP6	0,23	0,550	-0,98	**0,006**
TULP4	0,97	**0,003**	0,31	0,339
TWF2	-0,36	0,385	-1,06	**0,003**
TYROBP	0,07	0,799	-0,79	**0,005**
TYSND1	0,17	0,587	-1,30	**0,001**
U2AF1	0,71	0,094	-1,05	**0,003**
UBA1	0,44	0,271	-1,00	**0,007**
UBA2	0,50	0,151	1,05	**0,006**
UBA3	0,17	0,649	0,92	**0,002**
UBC	1,56	**0,001**	0,80	0,072
UBE2B	-0,48	0,139	1,18	**0,010**
UBE2Q2	0,72	**0,007**	0,23	0,473
UBE3C	1,30	**0,001**	0,29	0,489
UBE4B	1,19	**0,002**	0,17	0,705
UBR3	0,49	0,057	1,23	**0,000**
UBTD1	1,05	**0,005**	-0,21	0,545
UCHL1	0,23	0,432	-1,23	**0,000**
UCKL1	-1,33	**0,002**	-0,01	0,988
UCP2	-0,58	0,042	-1,01	**0,001**
UCP3	0,72	**0,009**	0,18	0,485
UGP2	-0,22	0,494	0,75	**0,010**
UHMK1	0,97	**0,004**	0,13	0,695
UHRF1BP1L	0,84	**0,010**	0,35	0,288
UNC13B	1,30	**0,000**	0,82	0,040
USP13	-0,69	0,118	1,11	**0,006**
USP2	-0,37	0,175	0,94	**0,008**
USP22	0,06	0,874	1,26	**0,001**

USP46	0,93	**0,003**	0,65	0,066
USP53	0,60	0,060	1,33	**0,000**
VAT1	0,13	0,768	-1,49	**0,000**
VCAM1	-0,04	0,922	-1,06	**0,001**
VCL	0,44	0,238	1,28	**0,000**
VEGFB	0,23	0,496	-0,96	**0,006**
VEZF1	1,13	**0,004**	0,47	0,296
VPRBP	-0,27	0,375	1,25	**0,003**
VWA1	-0,16	0,654	-1,34	**0,004**
WDR4	1,10	**0,007**	-0,28	0,400
WDR45	-0,16	0,660	0,93	**0,008**
WDR47	0,26	0,421	1,47	**0,000**
WNK1	-0,79	**0,009**	0,27	0,435
WRB	0,60	0,069	1,05	**0,004**
WSB1	-0,17	0,573	1,02	**0,003**
WTAP	0,31	0,300	1,13	**0,002**
WWP1	0,22	0,467	1,12	**0,009**
XIRP1	-0,85	**0,007**	-0,45	0,084
XIRP2	-0,07	0,812	1,03	**0,001**
XPO4	-0,93	**0,003**	-0,39	0,194
YME1L1	-0,05	0,880	1,00	**0,003**
YWHAE	0,16	0,568	1,52	**0,000**
ZAK	0,42	0,237	0,82	**0,007**
ZBTB16	0,49	0,094	1,89	**0,000**
ZBTB43	0,14	0,708	1,11	**0,005**
ZC3H12C	0,50	0,073	1,17	**0,004**
ZC3H14	-0,04	0,901	1,18	**0,003**
ZFAND6	-0,25	0,411	0,81	**0,007**
ZFP36	-0,44	0,132	0,73	**0,010**
ZFPM1	-0,04	0,900	-1,17	**0,005**
ZFPM2	0,47	0,111	0,90	**0,007**
ZFX	0,35	0,280	0,94	**0,006**
ZFYVE21	0,54	0,088	-1,18	**0,002**
ZFYVE26	-0,04	0,898	1,68	**0,003**
ZNF219	0,07	0,865	-1,15	**0,002**
ZNF32	0,55	0,240	1,02	**0,004**
ZNF341	0,34	0,367	1,17	**0,003**
ZNF503	-0,36	0,290	-0,96	**0,003**
ZNF524	0,14	0,591	-1,15	**0,008**
