# Supplementary material for: Skeletal Muscle Immunometabolism in Women With Polycystic Ovary Syndrome: A Meta-Analysis
Source: Front Physiol. 2020 Oct 22;11:573505. doi: 10.3389/fphys.2020.573505 (PMC7642984; doi:10.3389/fphys.2020.573505)
Supplement: Supplementary Table 3 — Overrepresentation analysis using gene ontology (GO) on the 114 genes differentially expressed in skeletal muscle from women with PCOS compared with controls (q < 0.05). [file Data_Sheet_1.PDF]

Supplemental Table 3. Overrepresentation analysis using gene ontology (GO) on the 114 genes differentially expressed in skeletal muscle from women with PCOS compared with controls ($q < 0.05$)

GO ID	GO Term	Size	%	*p*	Genes
GO:0016051	carbohydrate biosynthetic process	9	8,0	0,000	ALDOA, GPD2, PPP1R3B, PHKG1, UBC, BPGM, EGF, PGK1, PC
GO:0010648	negative regulation of cell communication	21	18,6	0,000	ING2, HFE2, MCL1, MSTN, RB1, RRM2B, THY1, CTNNB1, TRDN, FBXW7, AES, SERPINE2, SHISA2, PSMC4, ADM, UCP2, HMOX1, MAPKAP1, UBC, EGF, ACVR1
GO:0023057	negative regulation of signaling	21	18,6	0,000	ING2, HFE2, MCL1, MSTN, RB1, RRM2B, THY1, CTNNB1, TRDN, FBXW7, AES, SERPINE2, SHISA2, PSMC4, ADM, UCP2, HMOX1, MAPKAP1, UBC, EGF, ACVR1
GO:0009968	negative regulation of signal transduction	20	17,7	0,000	ING2, HFE2, MCL1, MSTN, RB1, RRM2B, CTNNB1, THY1, TRDN, FBXW7, AES, SERPINE2, SHISA2, PSMC4, ADM, HMOX1, MAPKAP1, UBC, EGF, ACVR1
GO:0008285	negative regulation of cell proliferation	15	13,3	0,000	COL4A3, HIST1H2AC, CTBP1, ING2, MSTN, RB1, ZBTB16, SKAP2, CTNNB1, FBXW7, ADM, SERPINE2, MORC3, HMOX1, ATOH8
GO:0048585	negative regulation of response to stimulus	22	19,5	0,000	ING2, HFE2, MCL1, BST2, MSTN, RB1, RRM2B, THY1, CTNNB1, TRDN, FBXW7, AES, SERPINE2, SHISA2, PSMC4, ADM, UCP2, HMOX1, MAPKAP1, UBC, EGF, ACVR1
GO:0051099	positive regulation of binding	7	6,2	0,000	PLCL1, AKTIP, RAN, RB1, EGF, PITX2, CTNNB1
GO:0006796	phosphate-containing compound metabolic process	35	31,0	0,000	ALDOA, LDHB, ATP5G2, CLK1, ENSA, RRM2B, CTNNB1, PLCL1, FBXW7, PPP1R3B, DUSP13, MORC3, H2AFY, FAM129A, EGF, INPP5A, PPTC7, GPD2, LPL, CTBP1, MOCS2, PHKG1, MYLK4, MSTN, BPGM, RB1, THY1, AKTIP, ADM, PSMC4, ADK, UBC, PGK1, SCP2, ACVR1
GO:0006793	phosphorus metabolic process	35	31,0	0,000	ALDOA, LDHB, ATP5G2, CLK1, ENSA, RRM2B, CTNNB1, PLCL1, FBXW7, PPP1R3B, DUSP13, MORC3, H2AFY, FAM129A, EGF, INPP5A, PPTC7, GPD2, LPL, CTBP1, MOCS2, PHKG1, MYLK4, MSTN, BPGM, RB1, THY1, AKTIP, ADM, PSMC4, ADK, UBC, PGK1, SCP2, ACVR1
GO:0051098	regulation of binding	9	8,0	0,001	PLCL1, AES, AKTIP, RAN, HMOX1, RB1, EGF, PITX2, CTNNB1
GO:0019725	cellular homeostasis	15	13,3	0,001	ALDOA, HFE2, MCL1, IREB2, MSTN, THY1, TRDN, ANXA7, ADM, UCP2, ATP2C1, HMOX1, ATP2A1, MT2A, UNC13B
GO:0010629	negative regulation of gene expression	21	18,6	0,001	HIST1H2AC, TSHZ2, CTBP1, HFE2, RAN, IFITM3, IREB2, RB1, ZBTB16, CTNNB1, ANXA7, TSC22D3, AES, SERPINE2, UBC, H2AFY, ATOH8, PTBP2, NFIA, PITX2, PC
GO:0010605	negative regulation of macromolecule metabolic process	28	24,8	0,001	HIST1H2AC, TSHZ2, HFE2, IFITM3, ZBTB16, ENSA, CTNNB1, ANXA7, TSC22D3, FBXW7, AES, SERPINE2, H2AFY, PTBP2, ATOH8, FAM129A, PITX2, COL4A3, CTBP1, BST2, RAN, IREB2, RB1, THY1, PSMC4, UBC, NFIA, PC
GO:0006094	gluconeogenesis	5	4,4	0,001	ALDOA, GPD2, BPGM, PGK1, PC
GO:0042592	homeostatic process	22	19,5	0,001	ALDOA, LPL, HFE2, MCL1, IREB2, MSTN, BPGM, RB1, HOMER1, CTNNB1, THY1, TRDN, ANXA7, TSC22D3, FBXW7, ADM, ATP2C1, UCP2, HMOX1, ATP2A1, MT2A, UNC13B
GO:0019319	hexose biosynthetic process	5	4,4	0,001	ALDOA, GPD2, BPGM, PGK1, PC
GO:0009892	negative regulation of metabolic process	29	25,7	0,001	HIST1H2AC, TSHZ2, HFE2, IFITM3, ZBTB16, ENSA, CTNNB1, ANXA7, TSC22D3, FBXW7, AES, SERPINE2, H2AFY, PTBP2, ATOH8, FAM129A, PITX2, COL4A3, CTBP1, BST2, RAN, IREB2, MSTN, RB1, THY1, PSMC4, UBC, NFIA, PC
GO:0010942	positive regulation of cell death	12	10,6	0,001	COL4A3, FBXW7, AES, ING2, MCL1, ADM, UCP2, HMOX1, UBC, ZBTB16, UNC13B, CTNNB1
GO:0046364	monosaccharide biosynthetic process	5	4,4	0,002	ALDOA, GPD2, BPGM, PGK1, PC
GO:0046916	cellular transition metal ion homeostasis	5	4,4	0,002	HFE2, ATP2C1, HMOX1, MT2A, IREB2
GO:0031324	negative regulation of cellular metabolic process	27	23,9	0,002	HIST1H2AC, TSHZ2, HFE2, IFITM3, ZBTB16, ENSA, CTNNB1, TSC22D3, FBXW7, AES, SERPINE2, H2AFY, PTBP2, ATOH8, FAM129A, PITX2, COL4A3, CTBP1, BST2, RAN, IREB2, MSTN, RB1, THY1, PSMC4, UBC, NFIA
GO:0060429	epithelium development	16	14,2	0,002	IRX3, VCL, CTNNB1, ANXA7, FRMD6, PSMC4, ADM, SERPINE2, RSPO3, UBC, H2AFY, ATOH8, PGK1, EGF, ACVR1, PITX2
GO:0051450	myoblast proliferation	3	2,7	0,002	MSTN, ATOH8, PITX2
GO:0016052	carbohydrate catabolic process	6	5,3	0,003	ALDOA, GPD2, PPP1R3B, PHKG1, BPGM, PGK1
GO:0043388	positive regulation of DNA binding	4	3,5	0,003	RB1, EGF, PITX2, CTNNB1
GO:0044275	cellular carbohydrate catabolic process	4	3,5	0,003	GPD2, PPP1R3B, PHKG1, BPGM
GO:0043065	positive regulation of apoptotic process	11	9,7	0,003	COL4A3, FBXW7, AES, ING2, MCL1, ADM, HMOX1, UBC, ZBTB16, UNC13B, CTNNB1
GO:0051145	smooth muscle cell differentiation	4	3,5	0,003	ADM, ACVR1, PITX2, CTNNB1
GO:0006090	pyruvate metabolic process	5	4,4	0,003	ALDOA, LDHB, BPGM, PGK1, PC
GO:0051101	regulation of DNA binding	5	4,4	0,003	HMOX1, RB1, EGF, PITX2, CTNNB1
GO:0023051	regulation of signaling	32	28,3	0,003	ING2, HFE2, MCL1, ENSA, RRM2B, SKAP2, CTNNB1, PLCL1, FBXW7, AES, SERPINE2, SHISA2, RSPO3, HMOX1, MAPKAP1, EGF, BST2, MSTN, RB1, HOMER1, THY1, TRDN, ADM, PSMC4, ATP2C1, UCP2, ATP2A1, UBC, USP46, UNC13B, GRB14, ACVR1
GO:0043068	positive regulation of programmed cell death	11	9,7	0,003	COL4A3, FBXW7, AES, ING2, MCL1, ADM, HMOX1, UBC, ZBTB16, UNC13B, CTNNB1
GO:0006875	cellular metal ion homeostasis	10	8,8	0,004	TRDN, ANXA7, HFE2, ADM, ATP2C1, HMOX1, MT2A, ATP2A1, IREB2, THY1
GO:0090279	regulation of calcium ion import	5	4,4	0,004	TRDN, EGF, HOMER1, CTNNB1, THY1
GO:0003012	muscle system process	9	8,0	0,004	TRDN, ALDOA, HSPB6, HMOX1, ATP2A1, MSTN, FBXO32, HOMER1, VCL
GO:0007519	skeletal muscle tissue development	6	5,3	0,004	TSC22D3, MSTN, RB1, HOMER1, PITX2, CTNNB1
GO:0001525	angiogenesis	9	8,0	0,004	COL4A3, ADM, RSPO3, HMOX1, EGF, ACVR1, PITX2, CTNNB1, THY1
GO:0010557	positive regulation of macromolecule biosynthetic process	20	17,7	0,004	PRR16, ING2, HFE2, RAN, MSTN, RB1, ZBTB16, CTNNB1, FBXW7, DCAF6, PSMC4, HSF2, HMOX1, UBC, ATOH8, EGF, FAM129A, NFIA, ACVR1, PITX2
GO:0031328	positive regulation of cellular biosynthetic process	21	18,6	0,005	PRR16, ING2, HFE2, RAN, MSTN, RB1, ZBTB16, CTNNB1, FBXW7, DCAF6, PSMC4, ADM, HSF2, HMOX1, UBC, ATOH8, EGF, FAM129A, NFIA, ACVR1, PITX2
GO:0010646	regulation of cell communication	31	27,4	0,005	ING2, HFE2, MCL1, ENSA, RRM2B, SKAP2, CTNNB1, PLCL1, FBXW7, AES, SERPINE2, SHISA2, RSPO3, HMOX1, MAPKAP1, EGF, BST2, MSTN, RB1, HOMER1, THY1, TRDN, ADM, PSMC4, ATP2C1, UCP2, UBC, USP46, UNC13B, GRB14, ACVR1
GO:0055082	cellular chemical homeostasis	12	10,6	0,005	TRDN, ANXA7, HFE2, ADM, UCP2, ATP2C1, HMOX1, MT2A, ATP2A1, IREB2, UNC13B, THY1
GO:0061138	morphogenesis of a branching epithelium	6	5,3	0,005	ADM, RSPO3, EGF, ACVR1, PITX2, CTNNB1
GO:0055076	transition metal ion homeostasis	5	4,4	0,005	HFE2, ATP2C1, HMOX1, MT2A, IREB2
GO:0060538	skeletal muscle organ development	6	5,3	0,005	TSC22D3, MSTN, RB1, HOMER1, PITX2, CTNNB1
GO:0002009	morphogenesis of an epithelium	10	8,8	0,005	IRX3, ADM, PSMC4, RSPO3, UBC, EGF, ACVR1, PITX2, CTNNB1, VCL
GO:0051172	negative regulation of nitrogen compound metabolic process	19	16,8	0,005	HIST1H2AC, TSHZ2, CTBP1, HFE2, RAN, IFITM3, IREB2, RB1, ZBTB16, CTNNB1, TSC22D3, FBXW7, AES, UBC, H2AFY, ATOH8, PTBP2, NFIA, PITX2
GO:0048878	chemical homeostasis	15	13,3	0,006	LPL, HFE2, IREB2, HOMER1, THY1, TRDN, ANXA7, FBXW7, ADM, UCP2, ATP2C1, HMOX1, ATP2A1, MT2A, UNC13B
GO:0007267	cell-cell signaling	19	16,8	0,006	BST2, CACNB1, STXBP3, ENSA, HOMER1, CTNNB1, PLCL1, AES, SERPINE2, PSMC4, ADM, SHISA2, UCP2, RSPO3, UBC, USP46, EGF, UNC13B, PITX2
GO:0009891	positive regulation of biosynthetic process	21	18,6	0,006	PRR16, ING2, HFE2, RAN, MSTN, RB1, ZBTB16, CTNNB1, FBXW7, DCAF6, PSMC4, ADM, HSF2, HMOX1, UBC, ATOH8, EGF, FAM129A, NFIA, ACVR1, PITX2
GO:0044712	single-organism catabolic process	13	11,5	0,006	GPD2, ALDOA, COL4A3, LPL, PHKG1, BPGM, PLCL1, PSMC4, HMOX1, PGK1, SCP2, OAT, INPP5A
GO:0010558	negative regulation of macromolecule biosynthetic process	18	15,9	0,007	HIST1H2AC, TSHZ2, CTBP1, HFE2, RAN, IFITM3, IREB2, RB1, ZBTB16, CTNNB1, TSC22D3, FBXW7, AES, UBC, H2AFY, ATOH8, NFIA, PITX2
GO:0007063	regulation of sister chromatid cohesion	3	2,7	0,007	H2AFY, RB1, CTNNB1
GO:0010033	response to organic substance	29	25,7	0,007	ING2, HFE2, MCL1, IFITM3, ENSA, RRM2B, CTNNB1, ANXA7, AES, SHISA2, HMOX1, PITX2, LPL, COL4A3, BST2, RAN, MSTN, STXBP3, RB1, HOMER1, ADM, PSMC4, UCP2, MT2A, UBC, USP46, FBXO32, UNC13B, ACVR1
GO:0006006	glucose metabolic process	6	5,3	0,007	ALDOA, GPD2, PPP1R3B, BPGM, PGK1, PC
GO:0000122	negative regulation of transcription from RNA polymerase II promoter	12	10,6	0,007	TSC22D3, TSHZ2, CTBP1, AES, HFE2, UBC, H2AFY, RB1, ZBTB16, NFIA, PITX2, CTNNB1
GO:0044262	cellular carbohydrate metabolic process	7	6,2	0,007	GPD2, PPP1R3B, PHKG1, ADK, UBC, BPGM, INPP5A

GO:0009967	positive regulation of signal transduction	18	15,9	0,007	ING2, BST2, MCL1, MSTN, SKAP2, HOMER1, CTNNB1, TRDN, FBXW7, PSMC4, ATP2C1, RSPO3, HMOX1, UBC, EGF, UNC13B, GRB14, ACVR1
GO:0010647	positive regulation of cell communication	19	16,8	0,007	ING2, BST2, MCL1, MSTN, SKAP2, HOMER1, CTNNB1, TRDN, FBXW7, SERPINE2, PSMC4, ATP2C1, RSPO3, HMOX1, UBC, EGF, UNC13B, GRB14, ACVR1
GO:0001763	morphogenesis of a branching structure	6	5,3	0,008	ADM, RSPO3, EGF, ACVR1, PITX2, CTNNB1
GO:0042692	muscle cell differentiation	8	7,1	0,008	RBM24, ADM, MSTN, RB1, HOMER1, ACVR1, PITX2, CTNNB1
GO:0023056	positive regulation of signaling	19	16,8	0,008	ING2, BST2, MCL1, MSTN, SKAP2, HOMER1, CTNNB1, TRDN, FBXW7, SERPINE2, PSMC4, ATP2C1, RSPO3, HMOX1, UBC, EGF, UNC13B, GRB14, ACVR1
GO:2000113	negative regulation of cellular macromolecule biosynthetic process	17	15,0	0,008	HIST1H2AC, TSHZ2, CTBP1, HFE2, RAN, IREB2, ZBTB16, RB1, CTNNB1, TSC22D3, FBXW7, AES, UBC, H2AFY, ATOH8, NFIA, PITX2
GO:0010628	positive regulation of gene expression	20	17,7	0,008	PRR16, ING2, HFE2, RAN, MSTN, RB1, ZBTB16, CTNNB1, FBXW7, DCAF6, PSMC4, HSF2, UBC, H2AFY, ATOH8, EGF, FAM129A, NFIA, ACVR1, PITX2
GO:0055065	metal ion homeostasis	10	8,8	0,008	TRDN, ANXA7, HFE2, ADM, ATP2C1, HMOX1, MT2A, ATP2A1, IREB2, THY1
GO:0006816	calcium ion transport	8	7,1	0,008	TRDN, ATP2C1, ATP2A1, CACNB1, EGF, HOMER1, CTNNB1, THY1
GO:0032269	negative regulation of cellular protein metabolic process	14	12,4	0,009	COL4A3, CTBP1, BST2, RAN, IREB2, ENSA, RB1, CTNNB1, THY1, PSMC4, SERPINE2, UBC, H2AFY, FAM129A
GO:0051924	regulation of calcium ion transport	6	5,3	0,009	TRDN, CACNB1, EGF, HOMER1, CTNNB1, THY1
GO:0051402	neuron apoptotic process	6	5,3	0,009	FBXW7, USP53, MCL1, HMOX1, RB1, CTNNB1
GO:0005975	carbohydrate metabolic process	12	10,6	0,009	ALDOA, GPD2, LDHB, PPP1R3B, PHKG1, ADK, UBC, BPGM, EGF, PGK1, INPP5A, PC
GO:1903508	positive regulation of nucleic acid-templated transcription	17	15,0	0,009	ING2, HFE2, RAN, MSTN, RB1, ZBTB16, CTNNB1, FBXW7, DCAF6, PSMC4, HSF2, UBC, ATOH8, EGF, NFIA, ACVR1, PITX2
GO:0045893	positive regulation of transcription, DNA-templated	17	15,0	0,009	ING2, HFE2, RAN, MSTN, RB1, ZBTB16, CTNNB1, FBXW7, DCAF6, PSMC4, HSF2, UBC, ATOH8, EGF, NFIA, ACVR1, PITX2
GO:0030003	cellular cation homeostasis	10	8,8	0,010	TRDN, ANXA7, HFE2, ADM, ATP2C1, HMOX1, MT2A, ATP2A1, IREB2, THY1
GO:0051253	negative regulation of RNA metabolic process	16	14,2	0,010	HIST1H2AC, TSHZ2, CTBP1, HFE2, IFITM3, ZBTB16, RB1, CTNNB1, TSC22D3, AES, UBC, H2AFY, ATOH8, PTBP2, NFIA, PITX2
GO:0031327	negative regulation of cellular biosynthetic process	18	15,9	0,010	HIST1H2AC, TSHZ2, CTBP1, HFE2, RAN, IFITM3, IREB2, RB1, ZBTB16, CTNNB1, TSC22D3, FBXW7, AES, UBC, H2AFY, ATOH8, NFIA, PITX2
GO:0012501	programmed cell death	21	18,6	0,010	COL4A3, ING2, MCL1, MSTN, RB1, RRM2B, ZBTB16, NCKAP1, CTNNB1, TSC22D3, USP53, FBXW7, AES, ADM, AKTIP, UCP2, HMOX1, ATP2A1, UBC, UNC13B, ACVR1
GO:0044724	single-organism carbohydrate catabolic process	5	4,4	0,010	ALDOA, GPD2, PHKG1, BPGM, PGK1
GO:2001234	negative regulation of apoptotic signaling pathway	6	5,3	0,010	ING2, MCL1, HMOX1, RRM2B, ACVR1, CTNNB1
GO:0035295	tube development	10	8,8	0,010	IRX3, ING2, ADM, SERPINE2, RB1, ATOH8, EGF, ACVR1, PITX2, CTNNB1
GO:0051186	cofactor metabolic process	8	7,1	0,010	ALDOA, LDHB, MOCS2, HMOX1, IREB2, BPGM, PGK1, PC
GO:0008283	cell proliferation	21	18,6	0,011	COL4A3, HIST1H2AC, CTBP1, ING2, BST2, MSTN, RB1, ZBTB16, CLK1, SKAP2, CTNNB1, ANXA7, FBXW7, SERPINE2, ADM, MORC3, HMOX1, PTBP2, ATOH8, EGF, PITX2
GO:0051241	negative regulation of multicellular organismal process	14	12,4	0,011	COL4A3, IRX3, BST2, MSTN, ZBTB16, CTNNB1, THY1, TSC22D3, FBXW7, ADM, SERPINE2, HMOX1, ATP2A1, UBC
GO:1902680	positive regulation of RNA biosynthetic process	17	15,0	0,011	ING2, HFE2, RAN, MSTN, RB1, ZBTB16, CTNNB1, FBXW7, DCAF6, PSMC4, HSF2, UBC, ATOH8, EGF, NFIA, ACVR1, PITX2
GO:0009141	nucleoside triphosphate metabolic process	7	6,2	0,011	ALDOA, MOCS2, ADK, ATP5G2, BPGM, RRM2B, PGK1
GO:0045934	negative regulation of nucleobase-containing compound metabolic process	17	15,0	0,011	HIST1H2AC, TSHZ2, CTBP1, HFE2, IFITM3, ZBTB16, RB1, CTNNB1, TSC22D3, FBXW7, AES, UBC, H2AFY, ATOH8, PTBP2, NFIA, PITX2
GO:0006873	cellular ion homeostasis	10	8,8	0,011	TRDN, ANXA7, HFE2, ADM, ATP2C1, HMOX1, MT2A, ATP2A1, IREB2, THY1
GO:0006915	apoptotic process	20	17,7	0,011	COL4A3, ING2, MCL1, RB1, RRM2B, ZBTB16, NCKAP1, CTNNB1, TSC22D3, USP53, FBXW7, AES, ADM, AKTIP, UCP2, HMOX1, ATP2A1, UBC, UNC13B, ACVR1
GO:0044283	small molecule biosynthetic process	9	8,0	0,011	ALDOA, GPD2, LPL, ADM, BPGM, PGK1, OAT, SCP2, PC
GO:0009890	negative regulation of biosynthetic process	18	15,9	0,012	HIST1H2AC, TSHZ2, CTBP1, HFE2, RAN, IFITM3, IREB2, RB1, ZBTB16, CTNNB1, TSC22D3, FBXW7, AES, UBC, H2AFY, ATOH8, NFIA, PITX2
GO:0048514	blood vessel morphogenesis	9	8,0	0,012	COL4A3, ADM, RSPO3, HMOX1, EGF, ACVR1, PITX2, CTNNB1, THY1
GO:0000271	polysaccharide biosynthetic process	4	3,5	0,012	PPP1R3B, PHKG1, UBC, EGF
GO:0007166	cell surface receptor signaling pathway	27	23,9	0,012	ING2, HFE2, MCL1, TSPAN3, IFITM3, CTNNB1, FBXW7, AES, SHISA2, SERPINE2, RSPO3, HMOX1, ATOH8, EGF, PITX2, COL4A3, BST2, MSTN, RB1, HOMER1, NCKAP1, THY1, PSMC4, MT2A, UBC, UNC13B, ACVR1
GO:2000017	positive regulation of determination of dorsal identity	2	1,8	0,012	ACVR1, CTNNB1
GO:0051173	positive regulation of nitrogen compound metabolic process	20	17,7	0,013	PRR16, ING2, HFE2, RAN, MSTN, RB1, ZBTB16, CTNNB1, FBXW7, DCAF6, PSMC4, ADM, HSF2, UBC, ATOH8, EGF, FAM129A, NFIA, ACVR1, PITX2
GO:0035116	embryonic hindlimb morphogenesis	3	2,7	0,013	ZBTB16, PITX2, CTNNB1
GO:0051248	negative regulation of protein metabolic process	14	12,4	0,014	COL4A3, CTBP1, BST2, RAN, IREB2, ENSA, RB1, CTNNB1, THY1, PSMC4, SERPINE2, UBC, H2AFY, FAM129A
GO:0001944	vasculature development	10	8,8	0,014	COL4A3, FBXW7, ADM, RSPO3, HMOX1, EGF, ACVR1, PITX2, CTNNB1, THY1
GO:2001252	positive regulation of chromosome organization	5	4,4	0,014	CTBP1, ING2, H2AFY, RB1, CTNNB1
GO:0051254	positive regulation of RNA metabolic process	17	15,0	0,014	ING2, HFE2, RAN, MSTN, RB1, ZBTB16, CTNNB1, FBXW7, DCAF6, PSMC4, HSF2, UBC, ATOH8, EGF, NFIA, ACVR1, PITX2
GO:0019318	hexose metabolic process	6	5,3	0,015	ALDOA, GPD2, PPP1R3B, BPGM, PGK1, PC
GO:0060341	regulation of cellular localization	12	10,6	0,015	TRDN, FBXW7, BST2, UCP2, HMOX1, H2AFY, RB1, EGF, SCP2, UNC13B, CTNNB1, THY1
GO:0042127	regulation of cell proliferation	18	15,9	0,016	COL4A3, HIST1H2AC, CTBP1, ING2, MSTN, RB1, ZBTB16, SKAP2, CTNNB1, FBXW7, SERPINE2, ADM, MORC3, HMOX1, ATOH8, PTBP2, EGF, PITX2
GO:0010959	regulation of metal ion transport	7	6,2	0,016	TRDN, SERPINE2, CACNB1, EGF, HOMER1, CTNNB1, THY1
GO:0009966	regulation of signal transduction	27	23,9	0,016	ING2, HFE2, MCL1, RRM2B, SKAP2, CTNNB1, FBXW7, AES, SHISA2, SERPINE2, RSPO3, HMOX1, MAPKAP1, EGF, BST2, MSTN, RB1, HOMER1, THY1, TRDN, ADM, PSMC4, ATP2C1, UBC, UNC13B, GRB14, ACVR1
GO:0048729	tissue morphogenesis	10	8,8	0,016	IRX3, ADM, PSMC4, RSPO3, UBC, EGF, ACVR1, PITX2, CTNNB1, VCL
GO:0070838	divalent metal ion transport	8	7,1	0,016	TRDN, ATP2C1, ATP2A1, CACNB1, EGF, HOMER1, CTNNB1, THY1
GO:1902679	negative regulation of RNA biosynthetic process	15	13,3	0,016	HIST1H2AC, CTBP1, TSHZ2, HFE2, IFITM3, ZBTB16, RB1, CTNNB1, TSC22D3, AES, UBC, H2AFY, ATOH8, NFIA, PITX2
GO:0072511	divalent inorganic cation transport	8	7,1	0,016	TRDN, ATP2C1, ATP2A1, CACNB1, EGF, HOMER1, CTNNB1, THY1
GO:0001569	patterning of blood vessels	3	2,7	0,017	ACVR1, PITX2, CTNNB1
GO:1905114	cell surface receptor signaling pathway involved in cell-cell signaling	9	8,0	0,017	AES, SHISA2, PSMC4, RSPO3, UBC, EGF, UNC13B, PITX2, CTNNB1
GO:0001701	in utero embryonic development	7	6,2	0,018	ADM, PSMC4, RSPO3, UBR3, ACVR1, PITX2, CTNNB1
GO:0008219	cell death	21	18,6	0,018	COL4A3, ING2, MCL1, MSTN, RB1, RRM2B, ZBTB16, NCKAP1, CTNNB1, TSC22D3, USP53, FBXW7, AES, ADM, AKTIP, UCP2, HMOX1, ATP2A1, UBC, UNC13B, ACVR1
GO:0005996	monosaccharide metabolic process	6	5,3	0,018	ALDOA, GPD2, PPP1R3B, BPGM, PGK1, PC
GO:2000015	regulation of determination of dorsal identity	2	1,8	0,019	ACVR1, CTNNB1
GO:1903378	positive regulation of oxidative stress-induced neuron intrinsic apoptotic signaling pathway	2	1,8	0,019	FBXW7, MCL1
GO:0055080	cation homeostasis	10	8,8	0,019	TRDN, ANXA7, HFE2, ADM, ATP2C1, HMOX1, MT2A, ATP2A1, IREB2, THY1
GO:2000060	positive regulation of protein ubiquitination involved in ubiquitin-dependent protein catabolic process	4	3,5	0,019	FBXW7, PSMC4, UBC, EGF
GO:0055114	oxidation-reduction process	13	11,5	0,019	ALDOA, GPD2, LDHB, CTBP1, PPP1R3B, PHKG1, HMOX1, UBC, RRM2B, C1ORF43, PGK1, SCP2, MSRB3
GO:0009749	response to glucose	5	4,4	0,020	COL4A3, LPL, UCP2, ENSA, UNC13B

GO:2000026	regulation of multicellular organismal development	19	16,8	0,020	COL4A3, IRX3, MSTN, RB1, ZBTB16, CTNNB1, THY1, TSC22D3, FBXW7, SERPINE2, PSMC4, ADM, RSPO3, HMOX1, UBC, H2AFY, ATOH8, EGF, ACVR1
GO:0045742	positive regulation of epidermal growth factor receptor signaling pathway	3	2,7	0,020	FBXW7, UBC, EGF
GO:0010468	regulation of gene expression	38	33,6	0,020	IRX3, HIST1H2AC, TSHZ2, PRR16, ING2, HFE2, IFITM3, ZBTB16, CLK1, MLF1, CTNNB1, ANXA7, TSC22D3, FBXW7, AES, SERPINE2, HSF2, HMOX1, H2AFY, PTBP2, ATOH8, FAM129A, EGF, PITX2, RBM24, CTBP1, RAN, IREB2, MSTN, RB1, DCAF6, PSMC4, UBC, NGDN, CHAF1B, NFIA, PC, ACVR1
GO:0019637	organophosphate metabolic process	14	12,4	0,020	ALDOA, GPD2, LPL, LDHB, MOCS2, ADM, ADK, BPGM, ATP5G2, RRM2B, EGF, PGK1, SCP2, INPP5A
GO:2000027	regulation of organ morphogenesis	6	5,3	0,021	FBXW7, PSMC4, RSPO3, UBC, ACVR1, CTNNB1
GO:0035137	hindlimb morphogenesis	3	2,7	0,021	ZBTB16, PITX2, CTNNB1
GO:1901186	positive regulation of ERBB signaling pathway	3	2,7	0,021	FBXW7, UBC, EGF
GO:0022402	cell cycle process	16	14,2	0,021	ING2, RAN, ENSA, RB1, CTNNB1, MLF1, CSPP1, FBXW7, NEDD1, DUSP13, PSMC4, UBC, H2AFY, EGF, CLTCL1, ACVR1
GO:0009746	response to hexose	5	4,4	0,022	COL4A3, LPL, UCP2, ENSA, UNC13B
GO:0044723	single-organism carbohydrate metabolic process	10	8,8	0,022	ALDOA, GPD2, PPP1R3B, PHKG1, UBC, BPGM, EGF, PGK1, INPP5A, PC
GO:0098771	inorganic ion homeostasis	10	8,8	0,022	TRDN, ANXA7, HFE2, ADM, ATP2C1, HMOX1, MT2A, ATP2A1, IREB2, THY1
GO:0035239	tube morphogenesis	7	6,2	0,022	IRX3, ADM, ATOH8, EGF, ACVR1, PITX2, CTNNB1
GO:0014706	striated muscle tissue development	7	6,2	0,022	TSC22D3, MSTN, RB1, HOMER1, ACVR1, PITX2, CTNNB1
GO:0045892	negative regulation of transcription, DNA-templated	14	12,4	0,024	HIST1H2AC, CTBP1, TSHZ2, HFE2, ZBTB16, RB1, CTNNB1, TSC22D3, AES, UBC, H2AFY, ATOH8, NFIA, PITX2
GO:0034284	response to monosaccharide	5	4,4	0,024	COL4A3, LPL, UCP2, ENSA, UNC13B
GO:1903223	positive regulation of oxidative stress-induced neuron death	2	1,8	0,025	FBXW7, MCL1
GO:0048133	male germ-line stem cell asymmetric division	2	1,8	0,025	ING2, ZBTB16
GO:0061325	morphogenesis	2	1,8	0,025	PITX2, CTNNB1
GO:0042078	germ-line stem cell division	2	1,8	0,025	ING2, ZBTB16
GO:0098728	germline stem cell asymmetric division	2	1,8	0,025	ING2, ZBTB16
GO:0048584	positive regulation of response to stimulus	21	18,6	0,025	LPL, ING2, BST2, MCL1, MSTN, HOMER1, SKAP2, NCKAP1, CTNNB1, THY1, TRDN, FBXW7, PSMC4, ATP2C1, RSPO3, HMOX1, UBC, EGF, UNC13B, GRB14, ACVR1
GO:2000058	regulation of protein ubiquitination involved in ubiquitin-dependent protein catabolic process	4	3,5	0,025	FBXW7, PSMC4, UBC, EGF
GO:0007528	neuromuscular junction development	3	2,7	0,027	CACNB1, PDZRN3, UNC13B
GO:0042981	regulation of apoptotic process	16	14,2	0,027	COL4A3, ING2, MCL1, MSTN, RRM2B, ZBTB16, CTNNB1, TSC22D3, FBXW7, AES, ADM, UCP2, HMOX1, UBC, UNC13B, ACVR1
GO:0070647	protein modification by small protein conjugation or removal	13	11,5	0,027	UBR3, PDZRN3, ZBTB16, CTNNB1, FBXW7, DCAF6, USP53, PSMC4, AKTIP, UBC, USP46, FBXO32, EGF
GO:0043123	signaling	5	4,4	0,027	BST2, ATP2C1, HMOX1, UBC, CTNNB1
GO:0016055	Wnt signaling pathway	8	7,1	0,027	AES, SHISA2, PSMC4, RSPO3, UBC, EGF, PITX2, CTNNB1
GO:0060537	muscle tissue development	7	6,2	0,028	TSC22D3, MSTN, RB1, HOMER1, ACVR1, PITX2, CTNNB1
GO:0198738	cell-cell signaling by wnt	8	7,1	0,028	AES, SHISA2, PSMC4, RSPO3, UBC, EGF, PITX2, CTNNB1
GO:0045935	positive regulation of nucleobase-containing compound metabolic process	18	15,9	0,028	ING2, HFE2, RAN, MSTN, RB1, ZBTB16, CTNNB1, FBXW7, DCAF6, PSMC4, ADM, HSF2, UBC, ATOH8, EGF, NFIA, ACVR1, PITX2
GO:0001568	blood vessel development	9	8,0	0,028	COL4A3, ADM, RSPO3, HMOX1, EGF, ACVR1, PITX2, CTNNB1, THY1
GO:0034101	erythrocyte homeostasis	4	3,5	0,028	HMOX1, IREB2, BPGM, RB1
GO:0031400	negative regulation of protein modification process	9	8,0	0,028	CTBP1, PSMC4, UBC, H2AFY, ENSA, RB1, FAM129A, CTNNB1, THY1
GO:0010608	posttranscriptional regulation of gene expression	8	7,1	0,028	RBM24, PRR16, PSMC4, RAN, UBC, IREB2, NGDN, FAM129A
GO:0009144	purine nucleoside triphosphate metabolic process	6	5,3	0,029	ALDOA, MOCS2, ADK, ATP5G2, BPGM, PGK1
GO:0019079	viral genome replication	4	3,5	0,029	CTBP1, BST2, IFITM3, NFIA
GO:0043067	regulation of programmed cell death	16	14,2	0,029	COL4A3, ING2, MCL1, MSTN, RRM2B, ZBTB16, CTNNB1, TSC22D3, FBXW7, AES, ADM, UCP2, HMOX1, UBC, UNC13B, ACVR1
GO:0000280	nuclear division	9	8,0	0,030	NEDD1, ING2, DUSP13, RAN, H2AFY, ENSA, RB1, EGF, CLTCL1
GO:0070997	neuron death	6	5,3	0,030	FBXW7, USP53, MCL1, HMOX1, RB1, CTNNB1
GO:0009719	response to endogenous stimulus	17	15,0	0,030	ING2, HFE2, RAN, MSTN, STXBP3, RB1, RRM2B, HOMER1, CTNNB1, ADM, SHISA2, UCP2, HMOX1, UBC, FBXO32, ACVR1, PITX2
GO:0097193	intrinsic apoptotic signaling pathway	6	5,3	0,031	FBXW7, ING2, MCL1, HMOX1, ATP2A1, RRM2B
GO:0050801	ion homeostasis	10	8,8	0,031	TRDN, ANXA7, HFE2, ADM, ATP2C1, HMOX1, MT2A, ATP2A1, IREB2, THY1
GO:0061061	muscle structure development	9	8,0	0,031	TSC22D3, RBM24, ADM, MSTN, RB1, HOMER1, ACVR1, PITX2, CTNNB1
GO:0032268	regulation of cellular protein metabolic process	23	20,4	0,031	COL4A3, CTBP1, PRR16, ING2, BST2, RAN, IREB2, MSTN, ENSA, RB1, CTNNB1, THY1, PLCL1, FBXW7, SERPINE2, PSMC4, AKTIP, UBC, NGDN, H2AFY, EGF, FAM129A, ACVR1
GO:1903507	negative regulation of nucleic acid-templated transcription	14	12,4	0,032	HIST1H2AC, CTBP1, TSHZ2, HFE2, ZBTB16, RB1, CTNNB1, TSC22D3, AES, UBC, H2AFY, ATOH8, NFIA, PITX2
GO:2001233	regulation of apoptotic signaling pathway	7	6,2	0,032	FBXW7, ING2, MCL1, HMOX1, RRM2B, ACVR1, CTNNB1
GO:0045840	positive regulation of mitotic nuclear division	3	2,7	0,032	H2AFY, RB1, EGF
GO:2000273	positive regulation of receptor activity	3	2,7	0,032	TRDN, FBXW7, EGF
GO:0060070	canonical Wnt signaling pathway	6	5,3	0,033	AES, PSMC4, RSPO3, UBC, EGF, CTNNB1
GO:0009132	nucleoside diphosphate metabolic process	4	3,5	0,033	ALDOA, BPGM, RRM2B, PGK1
GO:0043525	positive regulation of neuron apoptotic process	3	2,7	0,033	FBXW7, MCL1, CTNNB1
GO:0016310	phosphorylation	22	19,5	0,034	ALDOA, CTBP1, PHKG1, MYLK4, MSTN, BPGM, RB1, CLK1, THY1, CTNNB1, PLCL1, FBXW7, AKTIP, PSMC4, MORC3, ADK, UBC, H2AFY, FAM129A, PGK1, EGF, ACVR1
GO:0045055	regulated exocytosis	6	5,3	0,034	ALDOA, HMOX1, STXBP3, EGF, UNC13B, VCL
GO:0051246	regulation of protein metabolic process	24	21,2	0,035	COL4A3, CTBP1, PRR16, ING2, BST2, RAN, IREB2, MSTN, ENSA, RB1, CTNNB1, THY1, PLCL1, FBXW7, SERPINE2, PSMC4, AKTIP, HMOX1, UBC, NGDN, H2AFY, EGF, FAM129A, ACVR1
GO:0009743	response to carbohydrate	5	4,4	0,036	COL4A3, LPL, UCP2, ENSA, UNC13B
GO:0007167	enzyme linked receptor protein signaling pathway	12	10,6	0,036	COL4A3, FBXW7, ING2, HFE2, SHISA2, UBC, MSTN, ATOH8, EGF, ACVR1, CTNNB1, NCKAP1
GO:0005976	polysaccharide metabolic process	4	3,5	0,036	PPP1R3B, PHKG1, UBC, EGF
GO:0006879	cellular iron ion homeostasis	3	2,7	0,036	HFE2, HMOX1, IREB2
GO:0006701	progesterone biosynthetic process	2	1,8	0,037	ADM, SCP2
GO:0030032	lamellipodium assembly	3	2,7	0,038	ABLIM1, NCKAP1, VCL
GO:0044057	regulation of system process	8	7,1	0,039	TRDN, ADM, HSPB6, HMOX1, ATP2A1, CACNB1, MSTN, FBXO32
GO:0051641	cellular localization	24	21,2	0,040	BST2, RAN, ATP5G2, RB1, ZBTB16, THY1, VCL, CTNNB1, TRDN, ADPRHL1, NEDD1, FBXW7, FRMD6, AKTIP, MORC3, UCP2, HMOX1, ATP2A1, UBC, H2AFY, EGF, SCP2, CLTCL1, UNC13B
GO:0036211	protein modification process	33	29,2	0,040	ING2, ZBTB16, ENSA, CLK1, CTNNB1, ADPRHL1, PLCL1, USP53, FBXW7, PPP1R3B, DUSP13, MORC3, H2AFY, FAM129A, EGF, PPTC7, CTBP1, PHKG1, MYLK4, UBR3, MSTN, PDZRN3, RB1, THY1, DCAF6, EGFLAM, AKTIP, PSMC4, UBC, USP46, FBXO32, FKBP2, ACVR1
GO:0006464	cellular protein modification process	33	29,2	0,040	ING2, ZBTB16, ENSA, CLK1, CTNNB1, ADPRHL1, PLCL1, USP53, FBXW7, PPP1R3B, DUSP13, MORC3, H2AFY, FAM129A, EGF, PPTC7, CTBP1, PHKG1, MYLK4, UBR3, MSTN, PDZRN3, RB1, THY1, DCAF6, EGFLAM, AKTIP, PSMC4, UBC, USP46, FBXO32, FKBP2, ACVR1
GO:0048641	regulation of skeletal muscle tissue development	3	2,7	0,040	TSC22D3, MSTN, CTNNB1
GO:0070527	platelet aggregation	3	2,7	0,040	SERPINE2, STXBP3, VCL
GO:0006812	cation transport	12	10,6	0,041	TRDN, SERPINE2, UCP2, ATP2C1, ATP2A1, CACNB1, IREB2, ATP5G2, EGF, HOMER1, CTNNB1, THY1
GO:0048285	organelle fission	9	8,0	0,041	NEDD1, ING2, DUSP13, RAN, H2AFY, ENSA, RB1, EGF, CLTCL1

GO:0044092	negative regulation of molecular function	13	11,5	0,041	TRDN, COL4A3, AES, SERPINE2, BST2, PSMC4, HMOX1, UBC, MSTN, H2AFY, ENSA, RB1, THY1
GO:0046496	nicotinamide nucleotide metabolic process	4	3,5	0,042	ALDOA, LDHB, BPGM, PGK1
GO:0019362	pyridine nucleotide metabolic process	4	3,5	0,042	ALDOA, LDHB, BPGM, PGK1
GO:0009250	glucan biosynthetic process	3	2,7	0,042	PPP1R3B, PHKG1, UBC
GO:0002262	myeloid cell homeostasis	4	3,5	0,043	HMOX1, IREB2, BPGM, RB1
GO:0003008	system process	20	17,7	0,043	ABLIM1, ALDOA, COL4A3, CACNB1, IREB2, MSTN, UBR3, RRM2B, HOMER1, VCL, TRDN, USP53, SERPINE2, HSPB6, ADM, HMOX1, ATP2A1, FBXO32, OAT, UNC13B
GO:0036480	neuron intrinsic apoptotic signaling pathway in response to oxidative stress	2	1,8	0,043	FBXW7, MCL1
GO:1902177	positive regulation of oxidative stress-induced intrinsic apoptotic signaling pathway	2	1,8	0,043	FBXW7, MCL1
GO:0090092	regulation of transmembrane receptor protein serine/threonine kinase signaling pathway	5	4,4	0,044	ING2, HFE2, UBC, MSTN, ACVR1
GO:0060562	epithelial tube morphogenesis	6	5,3	0,044	IRX3, ADM, EGF, ACVR1, PITX2, CTNNB1
GO:0030858	positive regulation of epithelial cell differentiation	3	2,7	0,045	H2AFY, ATOH8, CTNNB1
GO:0009887	organ morphogenesis	12	10,6	0,045	ABLIM1, IRX3, FBXW7, AES, ADM, PSMC4, RSPO3, UBC, ACVR1, PITX2, CTNNB1, THY1
GO:0007062	sister chromatid cohesion	4	3,5	0,046	FBXW7, H2AFY, RB1, CTNNB1
GO:0030521	androgen receptor signaling pathway	3	2,7	0,046	RAN, RB1, CTNNB1
GO:0010941	regulation of cell death	16	14,2	0,048	COL4A3, ING2, MCL1, MSTN, RRM2B, ZBTB16, CTNNB1, TSC22D3, FBXW7, AES, ADM, UCP2, HMOX1, UBC, UNC13B, ACVR1
GO:0051785	positive regulation of nuclear division	3	2,7	0,048	H2AFY, RB1, EGF
GO:0048732	gland development	7	6,2	0,048	FBXW7, SERPINE2, UCP2, HMOX1, EGF, PITX2, CTNNB1
GO:0072524	pyridine-containing compound metabolic process	4	3,5	0,049	ALDOA, LDHB, BPGM, PGK1
GO:1903376	regulation of oxidative stress-induced neuron intrinsic apoptotic signaling pathway	2	1,8	0,049	FBXW7, MCL1
